# Cardiovascular Complications Associated with Uro-Oncology Treatments—A Primer for the Clinician

**DOI:** 10.3390/diagnostics16152452

**Published:** 2026-08-03

**Authors:** Diana-Ligia Pena, Adriana-Mihaela Ilieșiu, Justin Aurelian, Mihai Grigore, Andreea-Simona Hodorogea, Catalina Coriu-Georgescu, Emma Weiss, Elisabeta Badilă, Viorel Jinga, Ana-Maria Balahura

**Affiliations:** 1 Department of Cardiology, Carol Davila University of Medicine and Pharmacy, 050474 Bucharest, Romania; mihai.grigore@umfcd.ro (M.G.); andreea.hodorogea@umfcd.ro (A.-S.H.); dr.coriucatalina@gmail.com (C.C.-G.); emma.weiss@umfcd.ro (E.W.); elisabeta.badila@umfcd.ro (E.B.); ana-maria.balahura@umfcd.ro (A.-M.B.); 2 Department of Cardiology, “Prof. Dr. Theodor Burghele” Clinical Hospital, 061344 Bucharest, Romania; dianaligiapena@gmail.com; 3 “Prof. Dr. Theodor Burghele” Clinical Hospital, 061344 Bucharest, Romania; justin.aurelian@umfcd.ro (J.A.); viorel.jinga@umfcd.ro (V.J.); 4 Department of Urology, Carol Davila University of Medicine and Pharmacy, 050474 Bucharest, Romania; 5 Colentina Clinical Hospital, 020125 Bucharest, Romania

**Keywords:** bladder cancer, cancer therapy-related cardiovascular toxicity, uro-cardio-oncology, immunotherapy, prostate cancer, urological cancer treatment

## Abstract

Cardiovascular complications increasingly challenge survivors of urological cancers, given the cardiotoxicity of therapies such as androgen deprivation, vascular endothelial growth factor receptor inhibitors, tyrosine kinase inhibitors, immune checkpoint inhibitors, and chemotherapy. This narrative review addresses the complex crosstalk between urological cancer treatment and cardiovascular disease. It summarizes cardiovascular toxicities linked to major antineoplastic agents, explores underlying mechanisms including metabolic and immune-mediated effects, and proposes strategies for surveillance, diagnosis, and management. Highlighting the need for multidisciplinary collaboration, it outlines future directions for research to optimize cardiovascular outcomes in this high-risk population. The increasing complexity of cardiovascular care in patients with urological malignancies highlights the need for closer collaboration between cardiologists, urologists, and oncologists, with uro-cardio-oncology emerging as an important multidisciplinary field.

## 1. Introduction

Cardiovascular complications are increasingly recognized as a critical concern in the management of urological cancers, particularly prostate cancer, which represents the largest subset of uro-oncological malignancies globally [1]. Advances in cancer treatment, including androgen deprivation therapy (ADT), chemotherapy, and novel targeted agents, have significantly improved patient survival but carry a substantial risk of cardiovascular toxicities [2]. These toxicities span a wide spectrum including ischemic heart disease (IHD), heart failure (HF), arrhythmias, hypertension (HTN), myocarditis, and metabolic disturbances, presenting a complex challenge for clinicians managing this vulnerable population [3].

Importantly, the cardiovascular burden in uro-oncology is strongly influenced by the age distribution of these malignancies. Prostate and bladder cancers occur predominantly in older individuals, who frequently present with pre-existing cardiovascular disease, diabetes mellitus, HTN, dyslipidemia, chronic kidney disease, obesity, smoking history and frailty syndromes [4,5]. In contrast, testicular cancer typically affects younger patients [6]; therefore, cardiovascular risk in uro-oncology reflects not only treatment-related toxicity but also the interaction between aging, baseline comorbidities, and cancer-associated systemic inflammation, all of which may be exacerbated by anticancer therapies [7].

Consequently, cardiovascular complications in uro-oncology should be interpreted within three complementary clinical settings: baseline cardiovascular risk before cancer treatment, treatment-emergent cardiotoxicity, and long-term cardiovascular sequelae among cancer survivors.

ADT, a cornerstone of prostate cancer therapy, profoundly affects androgen levels essential for cardiovascular homeostasis, thereby increasing the incidence of adverse cardiovascular events [1,8]. Moreover, emerging agents targeting androgen synthesis and receptor pathways carry their own cardiovascular risk profiles that remain incompletely understood [2]. Other systemic treatments and radiation therapy also contribute to cardiovascular morbidity, underscoring the necessity for integrated cardiovascular risk assessment and personalized management in uro-oncology patients [9].

Cardiology evaluation before the treatment is strongly recommended in patients with established cardiovascular disease, multiple cardiovascular risk factors, previous exposure to potentially cardiotoxic cancer therapies or when initiating therapies associated with a high cardiovascular risk profile, including ADT, VEGF inhibitors, anthracyclines, immune checkpoint inhibitors and cisplatin-based chemotherapy [8,10].

In this narrative review, we aim to synthesize current evidence on cardiovascular complications linked to common urological cancer treatments, and explore underlying pathophysiological mechanisms, with a focus on raising awareness for early detection and prevention. Unlike previous reviews that primarily summarize cardiovascular adverse effects of individual therapies, this review integrates current uro-oncological treatment strategies with practical cardiovascular risk stratification, surveillance and management recommendations across the major urological malignancies.

## 2. Materials and Methods

A comprehensive literature search was conducted to identify relevant studies, reviews, and clinical guidelines addressing cardiovascular complications associated with treatments for urological cancers, including but not limited to prostate, bladder, kidney and testicular cancers.

Electronic databases, including PubMed, MEDLINE, Scopus, and Web of Science, were searched for English-language articles published up to May 2026, as part of a narrative literature review. An updated supplementary literature search was performed in July 2026 to capture references published after the initial May 2026 cutoff.

The search strategy combined keywords including “cardiovascular complications”, “uro-oncology”, “urological cancer treatment”, “cardiotoxicity”, and “cancer therapy-related cardiovascular toxicity”, with an additional targeted update search for key drug classes, including ADT, androgen receptor pathway inhibitors (ARPIs), immune checkpoint inhibitors (ICIs), and vascular endothelial growth factor (VEGF) inhibitors.

Articles considered for inclusion comprised original research, reviews, meta-analyses, and clinical guidelines reporting on cardiovascular risks, pathophysiological mechanisms, and clinical outcomes related to urological cancer therapies, including hormonal treatments, systemic therapies, and radiation. Given the narrative design of this review, articles were selected based on clinical relevance and topical coverage rather than a systematic, protocol-driven screening process; no formal PRISMA methodology, dual-reviewer screening, or risk-of-bias assessment was applied. Relevant data were qualitatively synthesized to provide a comprehensive, clinically oriented overview of cardiovascular complications across the spectrum of urological cancer treatments.

Generative artificial intelligence (GenAI) tools were used exclusively after completion of the literature review and manuscript drafting for language editing, grammar refinement, and improvement of readability. No GenAI tools were used for literature selection, data extraction, study screening, interpretation of evidence, synthesis of scientific content, or formulation of conclusions. All scientific decisions and final manuscript revisions were performed and verified by the authors.

## 3. Cancer Therapy-Related Cardiovascular Toxicity Definitions Applied Across Oncology Subspecialities

Cancer therapy-related cardiovascular toxicity (CTR-CVT) encompasses a broad spectrum of cardiac, vascular, and metabolic complications associated with modern cancer therapies. The 2022 European Society of Cardiology (ESC) Guidelines on Cardio-Oncology established standardized definitions for CTR-CVT to reduce heterogeneity in diagnosis, reporting, and management across oncology subspecialties, and these definitions remain the current framework in clinical practice [8].

Cancer therapy-related cardiac dysfunction (CTR-CD) is identified either by clear symptoms—ranging from mild HF symptoms to severe decompensated HF requiring hospitalization, inotropic support, or mechanical circulatory assistance—or by significant, measurable changes in heart function, such as a notable drop in left ventricular ejection fraction (LVEF), decline in strain measurements, or elevation in cardiac biomarkers, even when symptoms are absent [8]. These criteria are particularly relevant in patients receiving systemic uro-oncological therapies, where subclinical myocardial injury may precede overt HF.

Immune checkpoint inhibitor (ICI) myocarditis is diagnosed on biopsy by the presence of inflammatory infiltrates with myocyte necrosis, or through clinical assessment combining elevated troponin and specific imaging or symptom criteria. The condition’s severity, from fulminant presentations with cardiogenic shock, malignant ventricular arrhythmias, or severe hemodynamic instability requiring intensive care support, to mild or steroid-resistant cases, is determined by clinical presentation and response to therapy, while recovery is measured by improvement in symptoms, cardiac tests, and biomarkers [8]. Recent position papers and expert consensus documents have further emphasized the importance of early recognition, rapid diagnostic pathways, and multidisciplinary management of ICI-related myocarditis, given its high mortality despite its relatively low incidence [11].

Vascular toxicity includes both silent and clinical presentations, covering accelerated atherosclerosis, endothelial dysfunction, peripheral artery disease, arterial or venous thromboembolism (VTE) or vasospasm. These can be discovered by imaging tests or manifest as acute events like myocardial infarctions or strokes [8].

Cancer therapy-related arterial HTN is defined according to specific blood pressure (BP) thresholds, which account for patient risk; therapy may need to be paused or acutely managed when readings are severely elevated or organ damage is suspected [8].

Arrhythmias related to cancer-therapy comprise a heterogeneous group of rhythm disturbances, including atrial fibrillation (AF), atrial flutter, supraventricular tachycardias, sinus bradycardia, conduction disorders, ventricular arrhythmias and QT interval prolongation [12]. QT prolongation is stratified: values below 480 ms allow therapy continuation, 480–500 ms require caution, and above 500 ms warrants treatment interruption and comprehensive reassessment because of the increased risk of torsades de pointes and sudden cardiac death. Other arrhythmias, including bradycardia, supraventricular and ventricular tachycardia, and AF, are screened and managed per ESC current arrhythmia guidelines [8,13,14,15,16].

## 4. Cardiovascular Toxicity of Uro-Oncological Drug Classes and Radiotherapy

Cardiovascular toxicity has emerged as a major determinant of morbidity and mortality in patients undergoing treatment for urological malignancies. Contemporary uro-oncological therapies—including ADT, ARPis, targeted therapies, immunotherapy and radiotherapy—have substantially improved oncological outcomes and survival, but are increasingly associated with a broad spectrum of cardiovascular adverse events [8].

These toxicities range from metabolic disturbances, HTN, arrhythmias, and arterial and venous thromboembolic events to IHD, HF, myocarditis, and treatment-related vascular injury, and accelerated arterial aging [8]. For clarity, cardiovascular toxicities are discussed according to common clinical manifestations, underlying mechanisms, clinical relevance, and implications for cardiovascular surveillance before, during and after cancer treatment.

A comprehensive overview of the principal cardiovascular toxicities associated with major uro-oncological therapies is summarized in Table 1.

### 4.1. Androgen Deprivation Therapies (ADT) and Androgen Receptor Pathway Inhibitors (ARPIs)

ADT and ARPIs are cornerstones of treatment for advanced prostate cancer, including metastatic hormone-sensitive and castration-resistant disease, primarily by suppressing androgen synthesis or blocking androgen receptor signaling [17,60].

ADT includes GnRH (gonadotropin-releasing hormone) agonists and antagonists, whereas ARPIs are represented mainly by androgen biosynthesis inhibitors (such as abiraterone), which reduce androgen production, and androgen receptor antagonists (such as enzalutamide, apalutamide, darolutamide), which directly block the androgen receptors [17,60]. While these agents have significantly improved oncologic outcomes, growing evidence demonstrates that they are associated with a broad spectrum of cardiovascular toxicities, which substantially contribute to non-cancer morbidity and mortality [2,18]. Notably, cardiovascular disease represents the second leading cause of death in men with prostate cancer, underscoring the clinical relevance of therapy-related cardiovascular risk in this population [61]. From a cardio-oncology perspective, understanding the mechanisms, clinical manifestations, and agent-specific cardiovascular risks associated with ADT and ARPIs is essential to optimize diagnostic strategies, risk stratification, and long-term patient management.

In contemporary prostate cancer management, ADT is frequently combined with ARPIs, chemotherapy, or radiotherapy to improve oncological outcomes, particularly in metastatic and high-risk disease, although these combinations may further amplify cardiovascular toxicity.

Androgen deprivation therapy encompasses both surgical (bilateral orchiectomy) and medical approaches (GnRH agonists or antagonists, oral antiandrogens) used to suppress serum testosterone. While medical castration allows for intermittent therapy—pausing treatment temporarily to allow testosterone recovery—surgical castration is permanent. Surgical castration causes testosterone to drop to castrate levels within hours, whereas medical castration with GnRH agonists causes a temporary “testosterone surge” for several weeks before levels eventually decrease. Surgical castration is associated with a higher incidence of cardiac ischemic events and peripheral artery disease during the first 18 months compared with medical castration using GnRH agonists [21,22]. After this initial period, long-term cardiovascular complication rates and mortality outcomes are generally similar between the two approaches [23]. For this reason, surgical castration is generally less favored in older patients or those with pre-existing cardiovascular disease, given the immediate and permanent drop in testosterone. Among medical castration options, GnRH antagonists appear to be associated with a relatively lower risk of major adverse cardiovascular and cerebrovascular events compared with GnRH agonists [62].

- Metabolic effects and atherosclerotic risk:

ADT induces profound metabolic alterations and heightens cardiovascular risk. Testosterone depletion impairs lipid metabolism, leading to increased total cholesterol, low-density lipoprotein (LDL), and triglycerides, alongside reduced high-density lipoprotein (HDL) levels [17]. Additionally, ADT promotes insulin resistance and increased adiposity characterized by increased fat mass and loss of lean muscle [63,64]. These metabolic derangements contribute to the development of metabolic syndrome, which is prevalent in over half of patients receiving long-term ADT [65]. The altered adipocytokines profile, with increased leptin and decreased cardioprotective adiponectin, further exacerbates cardiac risk [17].

Insulin resistance has been mechanistically linked to androgen receptor (AR) signaling deficiency in hepatocytes, as demonstrated in animal models showing enhanced gluconeogenesis and reduced glycolysis under AR knockout conditions [17]. Dysregulated lipid metabolism in adipose tissues with excessive lipid deposition in non-adipose organs also contributes to systemic cardiovascular toxicity [17].

- Endothelial dysfunction and HTN:

ADT is implicated in endothelial dysfunction, a key early event in atherosclerosis and vascular disease development. Experimental studies in animal models show that gonadotropin-releasing hormone (GnRH) agonists induce upregulation of angiotensin II type 1 receptor (AT1R) and NADPH oxidase (NOX2), leading to oxidative stress and reduced nitric oxide bioavailability, which together impair endothelial function [66]. Comparatively, GnRH antagonists appear to exert less deleterious effects on vascular function [17]. These observations have prompted increasing interest in agent-specific cardiovascular risk profiles among ADT modalities, which will be discussed in detail below.

Clinically, ADT is associated with both new-onset HTN and worsening of pre-existing HTN, particularly in older patients with baseline cardiovascular risk factors [2,18]. HTN remains one of the most consistently reported cardiovascular toxicities associated with ADT and requires careful baseline evaluation, serial BP monitoring, and aggressive management of modifiable cardiovascular risk factors throughout the treatment [8].

Clinically, these mechanisms may contribute to accelerated arterial aging, defined as the premature development of an older arterial phenotype characterized by endothelial dysfunction, increased arterial stiffness, impaired vasodilation, and accelerated atherosclerosis [8]. This process may partly explain the increased incidence of hypertension and ischemic events observed during prolonged ADT.

- Cardiac remodeling and HF risk:

Emerging clinical data reveal that ADT contributes to adverse cardiac remodeling, including reduced LVEF and progression toward HF, particularly in patients with pre-existing cardiac disease [67]. Mechanisms may involve androgen deprivation-induced myocardial fibrosis and altered calcium handling in myocytes, and activation of pro-fibrotic signaling pathways within the myocardium [62]. Accordingly, HF incidence is increased in men undergoing prolonged ADT, highlighting the need for baseline cardiac function assessment prior to treatment [17].

Importantly, the development of HF in patients receiving ADT is likely multifactorial and may result not only from direct myocardial effects, but also from treatment-associated HTN, accelerated atherosclerosis, endothelial dysfunction, arterial stiffness, and metabolic syndrome [68].

- Fluid retention and pulmonary edema:

Certain androgen-targeting therapies, particularly androgen biosynthesis inhibitors used in combination with ADT, frequently provoke fluid retention through renal sodium and water reabsorption linked to aldosterone pathway modulation [9]. This fluid overload clinically manifests as systemic and pulmonary congestion, with pulmonary edema being a significant cause of morbidity, especially in patients with pre-existing cardiac or renal dysfunction [69,70]. These effects necessitate careful volume status assessment and therapeutic intervention to prevent exacerbation of HF symptoms.

- Arrhythmias and autonomic nervous system effects:

ADT influences autonomic nervous system regulation by increasing sympathetic tone and reducing parasympathetic activity, which predisposes patients to arrhythmias, including AF and other tachyarrhythmias [71]. The incidence of AF may be modestly elevated in ADT-treated populations, warranting clinical and electrocardiographic (ECG) monitoring in those with risk factors [72]. Moreover, ADT-induced hormonal changes lengthen cardiac repolarization, increasing the risk of QT prolongation and life-threatening ventricular arrhythmias like Torsades des Pointes [73]. Given these effects, baseline and periodic electrocardiographic assessment may be particularly valuable in patients receiving long-term ADT or combination hormonal therapies. Patients receiving concomitant QT-prolonging medications or presenting electrolyte abnormalities require closer electrocardiographic surveillance.

- Thromboembolic risk:

Increased prothrombotic states have been observed in ADT-treated men, characterized by elevated fibrinogen levels, increased platelet aggregation, and heightened VTE risk [74]. Population-based analyses report approximately twofold increased risk for deep vein thrombosis and pulmonary embolism within the first years of ADT initiation [74]. These findings support careful assessment of thromboembolic risk and individualized consideration of thromboprophylaxis when otherwise clinically indicated [75].

Given the heterogeneity of androgen-targeting therapies, the cardiovascular safety profile varies across specific drug subclasses and individual agents. Moreover, cardiovascular toxicity profiles differ substantially between traditional ADT agents and newer ARPIs [76]. While GnRH agonists and antagonists are predominantly associated with metabolic abnormalities, endothelial dysfunction, accelerated atherosclerosis, and thromboembolic risk secondary to profound testosterone suppression, ARPIs may additionally induce HTN, fluid retention, QT prolongation, arrhythmias, and HF through distinct off-target molecular and hormonal mechanisms [77]. Consequently, understanding class-specific cardiovascular adverse effects is essential for individualized cardiovascular risk stratification and treatment selection in contemporary cardio-oncology practice.

Therefore, cardiovascular surveillance should be individualized according to the specific androgen-targeting strategy, baseline cardiovascular profile and planned treatment duration.

#### 4.1.1. GnRH Agonists (Leuprolide, Goserelin)

(a) *Cardiovascular adverse effects and within-class differences*

GnRH (gonadotropin-releasing hormone) agonists, such as leuprolide and goserelin, remain widely used for the treatment of prostate cancer. Beyond effective testosterone suppression, this drug class is consistently associated with unfavorable cardiometabolic changes, including insulin resistance, dyslipidemia, and increased fat mass, which collectively promote atherosclerosis and increase cardiovascular risk [78,79].

Observational meta-analyses have shown that patients receiving GnRH agonists have a significantly increased risk of major adverse cardiovascular events (MACE—the composite of cardiac death, myocardial infarction, coronary revascularization, stroke, and hospitalization because of HF), with incidences reported as high as 4.8% within 12 months of treatment initiation, higher compared with the 2.9% incidence of MACE observed with GnRH antagonists in the same timeframe [80,81].

(b) *Mechanisms of cardiovascular toxicity*

The mechanisms are predominantly related to testosterone-deprivation-induced metabolic changes, endothelial dysfunction, and altered vascular homeostasis (Section 4.1).

(c) *Clinical significance and long-term cardiovascular risk*

Not all randomized controlled trials have consistently demonstrated increased cardiovascular mortality with GnRH agonists; nevertheless, trends toward an increased incidence of nonfatal cardiovascular events remain evident [82]. This discrepancy may reflect limitations in trial design, selection of lower-risk trial populations, and relatively short follow-up compared with real-world treatment exposure [83].

The clinical impact is likely greatest in patients with established cardiovascular disease or multiple cardiovascular risk factors, particularly during the first year of treatment.

(d) *Role of the cardio-oncologist and monitoring*

Patients with established coronary artery disease, HF, or multiple cardiovascular risk factors may benefit from closer cardiovascular surveillance during GnRH agonist therapy. Baseline assessment should include cardiovascular history, BP, and evaluation of cardiovascular risk factors. Serial BP and metabolic monitoring should be performed during treatment, while ECG, cardiac imaging, and biomarkers should be used according to baseline risk, abnormal findings, or cardiovascular symptoms. Routine cardiology consultation is not necessary for every patient receiving a GnRH agonist but should be considered in patients with established or unstable cardiovascular disease, high cardiovascular risk, abnormal baseline findings, or new cardiovascular symptoms [8].

#### 4.1.2. GnRH Antagonists (Degarelix, Relugolix)

(a) *Cardiovascular adverse effects and within-class differences*

GnRH antagonists, including degarelix and relugolix, represent an alternative to GnRH agonists for androgen deprivation in prostate cancer, which are characterized by immediate suppression of gonadotropins without the initial testosterone surge [80].

The phase III HERO trial evaluated relugolix versus leuprolide in 930 men with advanced prostate cancer and reported a lower incidence of MACE at 48 weeks with relugolix than with leuprolide (2.9% vs. 6.2%; HR 0.46; 95% CI 0.24–0.88) [19,84,85].

Clinically relevant arrhythmias and HF exacerbations were also reported less frequently with relugolix, although event rates were low and the trial was not specifically powered for these cardiovascular endpoints. In contrast, the PRONOUNCE trial, which compared degarelix with leuprolide in men with prostate cancer and established atherosclerotic cardiovascular disease, did not demonstrate a statistically significant difference in MACE between groups; nonetheless, interpretation was limited by early termination and insufficient statistical power [20].

HTN and thromboembolic events have been reported less frequently with GnRH antagonists than with some other forms of ADT, although they may still occur as a consequence of testosterone suppression [86].

Updated systematic reviews and meta-analyses integrating clinical-trial and observational data have reported heterogeneous findings. Some pooled analyses suggest a lower incidence of MACE with GnRH antagonists than with GnRH agonists. Real-world evidence remains mixed and may be influenced by confounding by indication, because antagonists may preferentially be prescribed to patients perceived to have a higher baseline cardiovascular risk [81,87,88].

No direct evidence currently establishes a clinically meaningful difference in cardiovascular safety between degarelix and relugolix.

(b) *Mechanisms of cardiovascular toxicity*

The mechanisms associated with testosterone deprivation (including metabolic alterations, endothelial dysfunction, autonomic and repolarization abnormalities, and prothrombotic changes) are described in Section 4.1. In contrast to GnRH agonists, GnRH antagonists achieve immediate testosterone suppression without an initial testosterone surge and appear to exert fewer deleterious effects on vascular function [80].

(c) *Clinical significance and long-term cardiovascular risk*

Overall, the available evidence suggests a more favorable cardiovascular profile for GnRH antagonists than for GnRH agonists, particularly in patients with pre-existing cardiovascular disease. Nevertheless, the discordance between HERO, PRONOUNCE, pooled analyses, and real-world studies means that a definitive cardiovascular superiority of the antagonist class has not been established. ADT selection should remain individualized and should consider oncological indication, baseline cardiovascular risk, previous cardiovascular events, treatment duration, and patient preference. Cardiovascular risk is most relevant during active and prolonged treatment. A distinct delayed cardiovascular toxicity specific to GnRH antagonists has not been established; long-term risk is primarily related to continued testosterone suppression, persistence of metabolic risk factors, and underlying cardiovascular disease [80].

(d) *Role of the cardio-oncologist and monitoring*

Monitoring requirements for GnRH antagonists are generally the same as for other forms of ADT. Baseline evaluation should include ECG, BP, weight, fasting glucose, and lipid profile. During treatment, BP and metabolic parameters should be reassessed every 3–6 months, with annual cardiovascular risk reassessment during prolonged therapy. Particular attention should be paid to patients with established atherosclerotic cardiovascular disease, HF, arrhythmias, diabetes, chronic kidney disease, or multiple cardiovascular risk factors. Routine transthoracic echocardiography (TTE) and cardiac biomarker surveillance are not required in all patients receiving GnRH antagonists. ECG, TTE, troponin, or natriuretic peptide testing should be guided by baseline cardiovascular risk, abnormal findings, concomitant QT-prolonging medication, or new cardiovascular symptoms. Routine cardiology consultation is not necessary for every patient; referral should be considered before the treatment in patients with high or very high cardiovascular risk, established or unstable cardiovascular disease, significant arrhythmias, abnormal baseline investigations, or recent cardiovascular events [8].

#### 4.1.3. First-Generation Antiandrogens (Flutamide, Bicalutamide, Nilutamide)

(a) *Cardiovascular adverse effects and within-class differences*

Compared with GnRH agonists, first-generation antiandrogens appear to have comparatively lower cardiovascular toxicity than medical castration strategies [24]. The available evidence does not establish clinically meaningful differences in cardiovascular risk among flutamide, bicalutamide, and nilutamide. Their principal differences relate to their overall, predominantly non-cardiovascular tolerability profiles rather than to clearly distinct cardiotoxicity profiles [24].

(b) *Mechanisms of cardiovascular toxicity*

First-generation antiandrogens act as competitive antagonists of the androgen receptor. Their comparatively lower cardiovascular toxicity is probably related to the absence of profound testosterone suppression and to the more limited metabolic and vascular effects observed with these agents compared with medical castration strategies [89].

(c) *Clinical significance and long-term cardiovascular risk*

First-generation antiandrogens, including flutamide, bicalutamide, and nilutamide, were historically used as monotherapy or in combination with ADT [90].

Nonetheless, older-generation non-steroidal antiandrogen monotherapy is inferior to medical or surgical castration in terms of overall survival, clinical progression, treatment failure, and treatment discontinuation due to adverse events, and is generally not recommended [90].

In metastatic hormone-sensitive prostate cancer, contemporary guidelines do not recommend routine long-term combination of first-generation antiandrogens with GnRH agonists. Their principal remaining role is short-term administration during initiation of a GnRH agonist to reduce the risk of clinical testosterone flare [90].

The three agents differ mainly in their non-cardiovascular toxicity profiles. Nilutamide is associated with impaired adaptation to darkness and other visual disturbances, while interstitial pneumonitis has been reported in approximately 2% of exposed patients in controlled clinical trials [91,92]. Flutamide is particularly limited by gastrointestinal toxicity and hepatotoxicity; in a randomized comparison, diarrhea occurred more frequently with flutamide than with bicalutamide, 26% vs. 12%, respectively [93].

Bicalutamide generally has the most favorable tolerability profile among the three, although gynecomastia and breast pain are recognized adverse effects, particularly when it is used as monotherapy [93].

Accordingly, the comparatively limited cardiovascular toxicity reported with first-generation antiandrogens should not be interpreted as a reason to prefer them over more effective guideline-recommended contemporary treatment strategies [90].

(d) *Role of the cardio-oncologist and monitoring*

Current cardio-oncology guidelines do not provide a separate cardiovascular surveillance schedule specifically for first-generation antiandrogens. When these agents are used briefly for testosterone-flare prevention or in combination with a GnRH agonist, cardiovascular surveillance should follow the monitoring pathway for the accompanying ADT strategy and should be individualized according to baseline cardiovascular risk [8,90].

Baseline evaluation should include a cardiovascular history, physical examination, blood-pressure measurement, assessment of conventional cardiovascular risk factors, and a 12-lead ECG [8].

In patients without established cardiovascular disease who are expected to receive ADT, SCORE2 or SCORE2-OP may be used according to age to support baseline cardiovascular risk stratification [8].

Routine echocardiography and cardiac-biomarker surveillance are not required for every patient receiving a first-generation antiandrogen. TTE, cardiac troponin, and natriuretic peptides should be considered selectively according to baseline cardiovascular risk, pre-existing cardiovascular disease, abnormal initial findings, the concomitant anticancer regimen, or the development of cardiovascular symptoms [8].

Cardio-oncology or cardiology referral is recommended for patients classified as having high or very high baseline cardiovascular toxicity risk. Patients at low cardiovascular risk may generally be followed by the oncology or urology team, with referral if treatment-related cardiovascular toxicity, new cardiovascular symptoms, or uncontrolled cardiovascular risk factors develop [8].

Additional ECG surveillance is appropriate in patients with a prolonged baseline QTc interval, relevant electrolyte abnormalities, concomitant QT-prolonging medications, syncope, palpitations, or other findings suggesting increased arrhythmic risk [8].

Drug-specific non-cardiovascular safety monitoring remains important. Serum transaminases should be measured before the treatment and monitored during therapy because all three agents may cause hepatotoxicity [91].

For nilutamide, baseline chest radiography is recommended and pulmonary-function testing may be considered because of the risk of interstitial pneumonitis [91]. Bicalutamide labeling also recommends close PT/INR monitoring when it is co-administered with coumarin anticoagulants [93].

#### 4.1.4. Second-Generation Antiandrogens (Enzalutamide, Apalutamide, Darolutamide)

(a) *Cardiovascular adverse effects and within-class differences*

Second-generation ARPIs, including enzalutamide, apalutamide, and darolutamide, have demonstrated superior efficacy across the prostate cancer disease spectrum but exhibit a distinctly higher cardiovascular risk profile compared with first generation agents. Pivotal randomized controlled trials such as PROSPER (enzalutamide), SPARTAN (apalutamide), and ARAMIS (darolutamide) were conducted specifically in non-metastatic castration-resistant prostate cancer [25]. More recent systematic reviews and meta-analyses published in 2024 and 2025 have quantified these risks across individual agents particularly when ARPIs are combined with conventional ADT [26,94].

A systematic review and meta-analysis of 15,842 patients reported that use of ARPIs significantly increased rates of acute myocardial infarction (OR 1.96), coronary artery disease (OR 8.33 for enzalutamide), angina, and high-grade HTN (OR 4.94 for enzalutamide) compared with controls [95].

Emerging data also link second-generation antiandrogens to arrhythmogenic risks, particularly AF, while QT interval prolongation risk appears minimal according to clinical trial monitoring but warrants continued vigilance [89,96,97].

(b) *Mechanisms of cardiovascular toxicity*

The general mechanisms of ARPI-related cardiovascular toxicity are described in Section 4.1 and include androgen-receptor blockade, treatment-associated HTN, metabolic and vascular effects, and potential arrhythmogenic mechanisms.

(c) *Clinical significance and long-term cardiovascular risk*

Cardiovascular events may occur during active treatment, particularly in patients receiving concomitant ADT or with pre-existing cardiovascular disease. QT prolongation appears uncommon in clinical trials, whereas HTN and AF require greater clinical attention [89,96,97]. Long-term cardiovascular risk after treatment discontinuation remains insufficiently defined.

(d) *Role of the cardio-oncologist and monitoring*

Initial evaluation should include cardiovascular history, BP, ECG, and evaluation of modifiable risk factors. BP, symptoms, and electrolytes should be monitored during treatment, while TTE and cardiac biomarkers should be reserved for patients with pre-existing cardiovascular disease, abnormal baseline findings, or new symptoms [98]. Cardiology referral is not required routinely but should be considered in high-risk or symptomatic patients.

#### 4.1.5. Androgen Synthesis Inhibitors (Abiraterone)

Androgen synthesis inhibitors, with abiraterone acetate as the prototypical representative, inhibit CYP-17 mediated androgen biosynthesis and are integral in treating metastatic castration-resistant and castration-sensitive prostate cancer.

(a) *Cardiovascular adverse effects*

Despite their survival benefits as demonstrated in pivotal studies such as LATITUDE, STAMPEDE, and COU-AA-301, abiraterone is associated with a notable cardiovascular toxicity profile [99,100,101]. Real-world comparative studies published in 2025 further suggest that, among older men with metastatic castration-resistant prostate cancer, abiraterone may be associated with a higher risk of MACE compared with enzalutamide, although residual confounding cannot be fully excluded [27,98].

Abiraterone, through mineralocorticoid excess, commonly causes HTN, fluid retention, and hypokalemia, elevating HF risk [102]. A meta-analysis including over 8000 patients confirmed that abiraterone increases the relative risk of all-grade cardiac toxicity (RR 1.36; 95% CI 1.13–1.64) and HTN (RR 1.98; 95% CI 1.62–2.43) compared with controls [103].

(b) *Mechanisms of cardiovascular toxicity*

Abiraterone-induced CYP17 inhibition suppresses cortisol synthesis and leads to compensatory adrenocorticotropic hormone (ACTH) elevation, resulting in secondary mineralocorticoid excess characterized by sodium and water retention, HTN, hypokalemia, and fluid overload [104]. For this reason, low-dose corticosteroids such as prednisone are routinely co-administered in order to suppress ACTH production and mitigate mineralocorticoid-related adverse effects [105].

(c) *Clinical significance*

In contemporary cohorts, serious adverse events during abiraterone therapy, including cardiovascular complications requiring hospitalization, remain relevant and may prompt treatment interruption in vulnerable patients [106].

Prior to the widespread adoption of abiraterone, historical androgen- targeting agents such as ketoconazole and cyproterone acetate were used for similar indications. Ketoconazole, a non-selective CYP450 inhibitor, was historically used off-label for rapid androgen suppression but has since been largely abandoned due to hepatotoxicity and a narrower therapeutic window [28]. Cyproterone acetate, a steroidal antiandrogen with additional progestogenic activity, is no longer frequently used given the availability of more effective agents with better safety profiles across multiple disease stages; its use today is largely confined to select clinical settings or regions with limited access to newer ARPIs [29].

(d) *Role of the cardio-oncologist and monitoring*

Baseline assessment should include BP, serum potassium, liver-function tests, body weight, and volume status. During treatment, BP and potassium should be monitored weekly during the first month and monthly thereafter, while liver-function tests should be assessed monthly during the first three months. TTE and cardiac biomarkers should be reserved for patients with pre-existing cardiovascular disease, abnormal baseline findings, or new cardiovascular symptoms [8]. Cardiology referral should be considered in patients with HF, uncontrolled HTN, recurrent hypokalemia, clinically significant fluid retention, or cardiovascular symptoms during treatment.

### 4.2. Cytotoxic Chemotherapeutic Agents

Chemotherapy remains a key modality for treating *various urological cancers*, *including advanced bladder*, *prostate*, *and renal cell carcinomas*. Importantly, several chemotherapeutic classes carry notable cardiovascular risks [107,108].

The cardiovascular toxicity, manifesting as left ventricular systolic dysfunction, HF, arrhythmias, HTN, and VTE, can be acute (during or immediately after therapy), subacute (weeks post-therapy), or chronic (months to years later) [109,110].

#### 4.2.1. Platinum-Based Agents (Cisplatin, Carboplatin, Oxaliplatin)

(a) *Cardiovascular adverse effects and within-class differences*

Platinum compounds, especially cisplatin, are widely used in uro-oncology.

Clinical manifestations of cisplatin-associated cardiovascular toxicity include HTN, myocardial ischemia, particularly coronary vasospasm and myocardial infarction, venous and arterial thromboembolism, and, rarely, Takotsubo cardiomyopathy [32,107]. Cases of AF occurring during or shortly after cisplatin infusion have also been reported [111,112].

Carboplatin is considered less cardiotoxic than cisplatin but may still cause HTN and arrhythmias [113,114]. Oxaliplatin-related cardiotoxicity is rare but has been associated with acute coronary syndromes and arrhythmias, including conduction block, often in the context of hypersensitivity or anaphylactic reactions [31,115].

(b) *Mechanisms of cardiovascular toxicity*

Cisplatin-associated cardiotoxicity arises mainly through oxidative stress, endothelial injury, and electrolyte abnormalities, particularly hypomagnesemia and hypokalemia, which predispose to ischemic events and arrhythmias [31,32].

Cisplatin-induced nephrotoxicity may further increase cardiovascular risk by promoting HTN, and electrolyte disturbances [116]. The mechanisms associated with carboplatin are thought to overlap with those of cisplatin but are generally less severe [113,114].

(c) *Clinical significance and long-term cardiovascular risk*

Vascular toxicity may occur during active treatment but also contributes to increased long-term coronary and cerebrovascular risk. Cisplatin has been associated with accelerated atherosclerosis and late-onset myocardial infarction or cerebrovascular events [117]. These long-term effects are particularly relevant in young survivors treated with cisplatin-based regimens and are discussed further in Section 4.2.5.

(d) *Role of the cardio-oncologist and monitoring*

Baseline assessment before cisplatin-based chemotherapy should include ECG, renal function, serum magnesium and potassium, with TTE reserved for patients with additional cardiovascular risk factors or pre-existing cardiovascular disease. Renal function and electrolytes should be assessed during each treatment cycle, while ECG and further cardiac investigations should be performed in patients who develop palpitations, chest pain, syncope, or other cardiovascular symptoms. Patients should also be monitored for arrhythmias during cisplatin infusion when clinically indicated [8].

Cardiology consultation is not required routinely for every patient but should be considered before the treatment in those with established cardiovascular disease, HF, significant arrhythmias, impaired renal function, or a high risk of fluid overload during hydration. Long-term survivors, particularly after cisplatin-based treatment for testicular cancer, require periodic assessment of BP, renal function, lipid profile, glucose, and other modifiable cardiovascular risk factors [8].

#### 4.2.2. Taxanes (Paclitaxel, Docetaxel, Cabazitaxel)

(a) *Cardiovascular adverse effects and within-class differences*

Meta-analyses indicate that taxanes induce cardiotoxicity in approximately 5% to 20% of patients [118], including left ventricular dysfunction, tachycardia, AF, conduction disorders, and ischemic events [119,120].

Paclitaxel has been associated particularly with transient bradycardia and conduction abnormalities, with reported incidences ranging from 0.1% to 31% [121].

Although uncommon, clinically significant docetaxel-associated fluid retention may precipitate HF decompensation in susceptible patients [122].

For cabazitaxel, contemporary syntheses of randomized controlled trials demonstrate a broadly consistent safety profile without unexpected new safety signals [37]. In the phase III CARD trial, which compared cabazitaxel with a switch to the alternative androgen receptor-targeted agent (abiraterone or enzalutamide) in patients previously treated with docetaxel, cardiac disorders were reported in 4.8% of cabazitaxel-treated patients vs. 0.8% in the AR-targeted therapy arm, likely reflecting a combination of direct drug effects and the comorbidity burden typical of heavily pretreated metastatic castration-resistant prostate cancer populations [36].

(b) *Mechanisms of cardiovascular toxicity*

Taxane-associated cardiovascular toxicity is primarily related to direct myocardial injury and arrhythmogenic effects [123]. Oxidative stress and genetic predisposition may further influence individual susceptibility.

(c) *Clinical significance and long-term cardiovascular risk*

Cardiac dysfunction may range from subclinical myocardial strain abnormalities to overt HF and conduction disorders [124].

In a breast-cancer cohort of 50 patients receiving paclitaxel, median LVEF decreased from 60% to 48% over 30 months [125].

Cardiovascular events associated with cabazitaxel should be interpreted in the context of the older, comorbid, and heavily pretreated metastatic castration-resistant prostate cancer population included in these studies.

(d) *Role of the cardio-oncologist and monitoring*

Baseline cardiovascular assessment should include cardiovascular history and ECG. ECG monitoring is particularly appropriate in patients with pre-existing conduction disease or concomitant QT-prolonging medication. TTE should be considered in patients with pre-existing HF, structural heart disease, abnormal baseline findings, or new cardiovascular symptoms. Cardiology referral is not routinely required but should be considered in patients with significant cardiac disease, conduction abnormalities, HF, or treatment-emergent cardiovascular symptoms [8].

#### 4.2.3. Anthracyclines (Doxorubicin)

(a) *Cardiovascular adverse effects*

Although anthracyclines are less frequently used in uro-oncology, including in selected aggressive bladder and prostate cancers, they warrant consideration because of their well-established risk of CTR-CD, dilated cardiomyopathy, and HF decompensation [110].

(b) *Mechanisms of cardiovascular toxicity*

Anthracycline-associated cardiotoxicity involves oxidative injury to cardiomyocytes, mitochondrial dysfunction, and apoptosis, with the risk increasing according to cumulative dose.

(c) *Clinical significance and long-term cardiovascular risk*

Cardiac dysfunction may progress even years after treatment, with a cumulative HF incidence of approximately 6% at 10 years and subclinical left ventricular (LV) dysfunction affecting up to 18% of treated patients [38,109].

(d) *Role of the cardio-oncologist and monitoring*

Baseline and serial assessment using LVEF and GLS, complemented by high-sensitivity troponin and NT-proBNP, in patients with increased baseline cardiovascular risk, abnormal initial findings, or new cardiovascular symptoms, may detect anthracycline-induced myocardial injury earlier than LVEF alone and facilitate timely intervention [126,127].

#### 4.2.4. Antimetabolites (5-Fluorouracil, Capecitabine)

(a) *Cardiovascular adverse effects*

Although more commonly used in gastrointestinal cancers, 5-fluorouracil (5-FU) and its prodrug capecitabine may occasionally be used in urogenital malignancies. These agents may cause coronary vasospasm, resulting in angina, myocardial infarction, arrhythmias, and, rarely, acute cardiomyopathy [39,107].

The incidence of symptomatic cardiotoxicity ranges from approximately 1% to 7.6% with higher rates reported in patients with pre-existing coronary artery disease [128,129].

(b) *Mechanisms of cardiovascular toxicity*

Proposed mechanisms include direct endothelial toxicity, coronary vasospasm, and mitochondrial damage in cardiomyocytes [107].

(c) *Clinical significance*

ECG changes such as ST-segment deviation and T-wave abnormalities are common during episodes of 5-FU-related cardiotoxicity [130], while rare cases of torsade de pointes have also been reported [131].

(d) *Role of the cardio-oncologist and monitoring*

Patients with pre-existing IHD require closer clinical and ECG surveillance. New chest pain, ischemic ECG changes, or arrhythmias during treatment should prompt immediate cardiovascular assessment.

#### 4.2.5. Chemotherapy for Testicular and Penile Cancer and Long-Term Cardiovascular Effects

Testicular cancer, though relatively rare, predominantly affects young men and carries a cure rate exceeding 95% with modern combination chemotherapy. Standard regimens are cisplatin-based combinations—BEP (bleomycin, etoposide, cisplatin), EP (etoposide, cisplatin), and VIP/VeIP (etoposide or vinblastine, ifosfamide, cisplatin) [132]. Penile cancer is more commonly treated with TIP (paclitaxel, ifosfamide, cisplatin), the current reference neoadjuvant regimen for bulky nodal disease, while TPF (taxane, cisplatin, 5-fluorouracil) offers comparable efficacy with higher toxicity, and PF (cisplatin, 5-fluorouracil) or carboplatin–taxane doublets remain pragmatic alternatives for less fit patients [133].

(a) *Cardiovascular adverse effects*

Cisplatin-based combinations are associated with acute arterial and venous thromboembolic events, ischemic complications, and Raynaud’s phenomenon. Their cardiovascular profiles may also reflect the accompanying agents: taxane-containing regimens may add arrhythmic or myocardial toxicity, whereas 5-fluorouracil-containing regimens may increase the risk of coronary vasospasm [33,34].

(b) *Mechanisms of cardiovascular toxicity*

Cisplatin causes direct endothelial injury and promotes a prothrombotic state, early atherosclerosis, and adverse metabolic changes, including weight gain, insulin resistance, and dyslipidemia [33,34]. Post-treatment renal impairment may further increase cardiovascular risk through HTN, metabolic abnormalities, and chronic kidney disease.

(c) *Clinical significance and long-term cardiovascular risk*

Despite excellent oncologic outcomes, these curative regimens expose young survivors to a 1.4- to 7-fold higher long-term risk of cardiovascular disease compared with the general population—a phenomenon often described as “accelerated vascular aging,” although data beyond 20 years of follow-up remain limited [34].

In a study assessing vascular stiffness via carotid-femoral pulse wave velocity in testicular cancer survivors treated with cisplatin-based chemotherapy versus orchiectomy alone and age-matched healthy controls, Stelwagen et al. demonstrated that very long-term survivors (median follow-up of 28 years) treated with cisplatin-based chemotherapy show increased vascular damage compatible with accelerated vascular aging, supporting the need for intensive long-term cardiovascular risk management [33].

In terms of long-term outcomes, survivors present a significantly elevated risk of myocardial infarction (2- to 3-fold compared with the general population in those treated with BEP) and angina [34]. Later studies show that even mild reductions in renal function post-chemotherapy signal elevated later cardiovascular risk [35].

(d) *Role of the cardio-oncologist and monitoring*

Baseline assessment should include cardiovascular risk factors, BP, renal function, and electrolytes, with additional cardiac testing guided by baseline risk.

Given this well-documented long-term risk, cardiovascular risk assessment and modifiable risk factor management (BP, lipids, glycemic control, smoking cessation) should be incorporated into routine long-term follow-up of testicular and penile cancer survivors, independent of oncologic surveillance [34].

Although testicular cancer accounts for only a small proportion of uro-oncological malignancies, it deserves particular attention because affected patients are typically young and have an excellent long-term survival. Consequently, cardiovascular prevention becomes an integral component of survivorship care, as cisplatin-based chemotherapy accelerates vascular ageing and increases the lifelong risk of myocardial infarction, metabolic syndrome and cardiovascular mortality.

### 4.3. Targeted Therapies and Novel Agents

Targeted therapies refer to anticancer agents designed to selectively inhibit molecular pathways involved in tumor growth, angiogenesis, proliferation, and metastatic progression, whereas the term novel agents encompasses recently developed therapies with distinct molecular targets or mechanisms of action that have expanded therapeutic options in uro-oncology [43,134].

The incorporation of these therapies has markedly improved the management of advanced urological malignancies, including renal *cell carcinoma*, *metastatic urothelial carcinoma*, *and castration-resistant prostate cancer*. Despite their therapeutic benefits, these agents are linked to distinct cardiovascular toxicities due to their specific modes of action on cellular signaling pathways and vascular homeostasis, necessitating careful monitoring and management [43,134].

#### 4.3.1. VEGF/VEGFR Inhibitors and Other Tyrosine Kinase Inhibitors (TKIs—Sunitinib, Pazopanib, Sorafenib, Axitinib, Cabozantinib, Lenvatinib, Tivozanib, Erdafitinib, Dovitinib and Derazantinib)

(a) *Cardiovascular adverse effects and within-class differences*

The VEGF/VEGFR (vascular endothelial growth factor) inhibitors, including sunitinib, pazopanib, sorafenib, axitinib, cabozantinib, lenvatinib, and tivozanib, are widely used in renal cell carcinoma and selected uro-oncological settings [43].

HTN is the most prevalent class-related cardiovascular toxicity and has been reported in up to 80% of patients [135,136].

Lenvatinib is associated with a particularly high HTN burden, with all-grade HTN reported in 50% and severe HTN in 14% of patients, while in patients receiving tivozanib severe HTN has been reported in up to 20% [42,43].

Other reported toxicities include arterial stiffness, LV hypertrophy, HF, arterial and venous thromboembolism, QT prolongation, and arrhythmias, the latter being particularly described with sunitinib [43].

Among non-VEGFR TKIs, erdafitinib is approved for FGFR-altered urothelial carcinoma and is associated mainly with hyperphosphatemia, HTN, and infrequent cardiac events [44]. Debio-1347, dovitinib and derazantinib remain investigational, and their cardiovascular toxicity profiles are insufficiently characterized, although HTN and cardiac dysfunction have been reported or are biologically plausible [45,137].

(b) *Mechanisms of cardiovascular toxicity*

VEGF-pathway inhibition reduces endothelial nitric oxide production, causing vasoconstriction, increased peripheral vascular resistance, and HTN [135,136].

Endothelial dysfunction and vascular injury increase arterial stiffness and afterload, thereby predisposing to LV hypertrophy, HF, accelerated atherosclerosis, and thromboembolic events [68].

Non-VEGFR TKIs may additionally induce mitochondrial dysfunction, oxidative stress, and direct cardiomyocyte injury, with toxicity varying according to kinase selectivity [138].

FGFR (Fibroblast growth factor receptor) inhibition may cause hyperphosphatemia through impaired renal phosphate excretion, potentially promoting vascular calcification [44].

(c) *Clinical significance and long-term cardiovascular risk*

HTN may develop rapidly, including during the first days of treatment, and may correlate with the degree of VEGF-pathway inhibition and treatment efficacy [135,136].

Anti-VEGF therapy may also contribute to arterial ischemic events and accelerated atherosclerosis, supporting continued management of global vascular risk during treatment [68].

Most cardiovascular toxicities improve following antihypertensive treatment, dose reduction, or treatment interruption, although long-term vascular consequences remain incompletely defined.

(d) *Role of the cardio-oncologist and monitoring*

Baseline assessment should include BP, ECG with QTc, and TTE/LVEF. Home BP monitoring is recommended weekly during the first treatment cycle and periodically thereafter, with early optimization of antihypertensive therapy. LVEF should be reassessed after approximately three months or earlier if cardiovascular symptoms develop. Cardiology referral and dose modification should be considered in patients with uncontrolled HTN, HF, significant QT prolongation, or other severe cardiovascular toxicity [139]. Because VEGF-pathway inhibition itself induces hypertension and may blunt the efficacy of concomitant antihypertensive agents, proactive dose titration and close home BP monitoring are recommended at treatment initiation and after any dose change [139].

#### 4.3.2. mTOR Inhibitors (Everolimus, Temsirolimus)

mTOR (mechanistic target of rapamycin) inhibitors, primarily everolimus and temsirolimus, are approved for the treatment of advanced renal cell carcinoma.

(a) *Cardiovascular adverse effects and within-class differences*

HTN is the most frequently reported cardiovascular adverse event, occurring in up to 24% of patients, while HF and LV dysfunction have been reported in up to 17% of everolimus-treated patients [47,140].

In contemporary practice, everolimus is frequently combined with lenvatinib, a regimen associated with HTN in approximately 42% of patients and severe HTN in about 13% [141].

(b) *Mechanisms of cardiovascular toxicity*

mTOR inhibitors may affect cardiovascular health by altering glucose metabolism, promoting insulin resistance and dyslipidemia, and impairing endothelial function, thereby contributing to HTN and vascular stiffness [46].

(c) *Clinical significance and long-term cardiovascular risk*

Cardiovascular toxicity is usually treatment-emergent and is particularly relevant when everolimus is combined with lenvatinib, where early and clinically significant HTN may lead to dose modification or treatment interruption [141].

(d) *Role of the cardio-oncologist and monitoring*

Baseline and serial BP monitoring is recommended, particularly during lenvatinib–everolimus therapy. Cardiac assessment should be guided by baseline cardiovascular risk or symptoms suggestive of LV dysfunction or HF [141].

#### 4.3.3. HIF-2α Inhibitors (Belzutifan)

Belzutifan, an HIF-2α (hypoxia-inducible factor 2-alpha) inhibitor, is approved for selected renal cell carcinoma indications. It has demonstrated durable antitumor activity, including an overall response rate of 77% in renal tumors in the relevant study population [111]. In the phase III LITESPARK-005 trial, belzutifan improved progression-free survival and objective response rate compared with everolimus in patients with advanced renal cell carcinoma previously treated with PD-1/PD-L1 inhibitors and VEGF-targeted therapy [48].

(a) *Cardiovascular adverse effects and within-class differences*

The adverse effects of belzutifan are dominated by anemia and hypoxia. Anemia has been reported in approximately 80–90% of patients, while hypoxia occurs in approximately 10–15% in contemporary phase II and III studies [48].

Earlier-phase studies reported clinically significant hypoxemia in up to 29% of patients, sometimes requiring supplemental oxygen or treatment interruption [142].

Overt cardiovascular toxicity, including HF or arrhythmias, has not been commonly reported, suggesting a relatively low direct cardiovascular toxicity profile [143].

(b) *Mechanisms of cardiovascular toxicity*

HIF-2α inhibition disrupts physiological oxygen-sensing pathways. Hypoxia may result from suppression of hypoxia-induced vasoconstriction, potentially causing ventilation–perfusion mismatch, while anemia further reduces systemic oxygen delivery [142].

(c) *Clinical significance and long-term cardiovascular risk*

Anemia and hypoxia may become clinically relevant during active treatment and may require transfusion, oxygen supplementation, dose interruption, or other supportive measures [48]. Current evidence does not suggest a distinct long-term cardiovascular toxicity syndrome.

(d) *Role of the cardio-oncologist and monitoring*

Baseline and periodic hemoglobin assessment and oxygen-saturation monitoring are recommended [48]. Cardiology evaluation should be symptom-driven or considered in patients with pre-existing cardiopulmonary disease or unexplained dyspnea, chest pain, arrhythmias, or signs of HF [48].

#### 4.3.4. PARP Inhibitors (Olaparib, Rucaparib, Talazoparib, Niraparib)

Poly (ADP-ribose) polymerase (PARP) inhibitors such as olaparib and rucaparib, have been approved for metastatic castration-resistant prostate cancer with DNA repair deficiencies, such as mutations in BRCA1 and BRCA2 genes [50].

(a) *Cardiovascular adverse effects and within-class differences*

Meta-analyses show an increase in MACE with an incidence of 5.0% in PARPi-treated patients vs. 3.6% in controls (Peto OR 1.62, *p* = 0.0009) [50]. HTN is the most common cardiovascular toxicity, occurring in 17.5% of patients, while thromboembolic events are increased with PARPi therapy but remain infrequent, apart from VTE (RR 2.17, *p* = 0.01) [144]. Available evidence does not yet establish clear differences in cardiovascular toxicity among individual PARP inhibitors.

(b) *Mechanisms of cardiovascular toxicity*

Their cardiotoxic mechanisms may involve oxidative stress, endothelial dysfunction, and interference with autonomic regulation and ion channels, but remain incompletely characterized [50,145].

(c) *Clinical significance and combination therapy*

PARP inhibitors are increasingly combined with androgen receptor pathway inhibitors, including olaparib plus abiraterone/prednisone, niraparib plus abiraterone/prednisone, and talazoparib plus enzalutamide, raising the possibility of additive cardiovascular toxicity. In the phase III PROpel trial, cardiovascular event rates did not differ significantly between olaparib–abiraterone and placebo–abiraterone, contrasting with an earlier, smaller phase II study that suggested an imbalance. Nevertheless, thromboembolic events, including pulmonary embolism, have been reported during combination therapy and remain relevant in patients with additional prothrombotic risk factors [30].

(d) *Role of the cardio-oncologist and monitoring*

Baseline assessment should include BP and evaluation of cardiovascular and thromboembolic risk factors. During treatment, BP and symptoms suggestive of arterial or venous thrombosis should be monitored, with cardiology assessment guided by baseline risk or treatment-emergent cardiovascular events [30].

#### 4.3.5. Antibody-Drug Conjugates (Enfortumab Vedotin)

Antibody-drug conjugates (ADCs) have become important therapies in urothelial carcinoma. Enfortumab vedotin is currently used both as monotherapy in previously treated disease and in combination with pembrolizumab in the first-line setting [146,147,148].

Enfortumab vedotin retains FDA approval in this setting, whereas sacituzumab govitecan’s accelerated approval for urothelial carcinoma was voluntarily withdrawn by the manufacturer in November 2024 after the confirmatory TROPiCS-04 trial failed to demonstrate an overall survival benefit; it remains approved only for breast cancer indications [146,147,148].

(a) *Cardiovascular adverse effects and within-class differences*

Despite their targeted nature, ADCs have been linked to significant cardiovascular toxicities. Among 1863 cardiovascular adverse events reported in FAERS (FDA Adverse Event Reporting System) database, the most frequent were HF (*n* = 277), and embolic and thrombotic venous events (*n* = 446) [51].

HTN and arrhythmias have also been reported, although a consistent direct cardiotoxicity signal specific to enfortumab vedotin has not been established [149].

(b) *Mechanisms of cardiovascular toxicity*

The exact mechanism of ADC-associated cardiotoxicity remain incompletely understood, but endothelial injury, pro-thrombotic states, and off-target cytotoxic effects likely contribute [150].

(c) *Clinical significance*

Enfortumab vedotin plus pembrolizumab is a standard first-line treatment for locally advanced or metastatic urothelial carcinoma. In the phase III EV-302 trial, grade ≥ 3 treatment-related adverse events occurred in 57.3% of patients receiving the combination vs. 69.5% receiving platinum-based chemotherapy, without a specific increase in direct cardiovascular toxicity [52]. Nevertheless, the combination includes the immune-mediated myocarditis risk associated with pembrolizumab, as discussed in Section 4.4.1.

(d) *Role of the cardio-oncologist and monitoring*

Cardiovascular assessment should be guided by baseline risk and symptoms. In patients receiving enfortumab vedotin plus pembrolizumab, new chest pain, dyspnea, palpitations, syncope, or suspected myocarditis should prompt ECG, troponin, and cardiac imaging according to the ICI monitoring pathway [52].

#### 4.3.6. Radioligand Therapy (Radium-223, ^177^Lu-PSMA-617)

(a) *Cardiovascular adverse effects*

Radium-223, an alpha-emitting radiopharmaceutical targeting bone metastases, demonstrated a favorable cardiovascular safety profile in the pivotal ALSYMPCA trial, with low rates of serious cardiac adverse events (myocardial infarction ~0.2%, atrial fibrillation ~0.4%) and no major safety signals identified during 3-year long-term follow-up [53].

Real-world pharmacovigilance data suggest that older patients (>85 years) may be at increased risk of cardiac adverse events compared with younger patients, and combination with abiraterone or enzalutamide has been associated with higher overall serious adverse event rates than radium-223 monotherapy [1].

^177^Lu-PSMA-617, a beta-emitting PSMA-targeted radioligand approved based on the VISION trial, has a safety profile dominated by hematologic and renal toxicity (xerostomia, cytopenias, nephrotoxicity from off-target renal PSMA expression) rather than direct cardiotoxicity; no major cardiac safety signal has been reported to date, though dedicated long-term cardiovascular follow-up data remain limited [54].

(b) *Mechanisms of cardiovascular toxicity*

No specific direct myocardial toxicity mechanism has been established. Clinically relevant toxicity is predominantly related to radiation-induced bone-marrow suppression and, for ^177^Lu-PSMA-617, renal exposure and nephrotoxicity [1].

(c) *Clinical significance and long-term cardiovascular risk*

Cardiovascular events appear uncommon during treatment, although dedicated long-term cardiovascular follow-up remains limited. In older and comorbid patients, reported cardiovascular events should be interpreted in the context of baseline cardiovascular disease and concomitant androgen-targeted therapy [1].

(d) *Role of the cardio-oncologist and monitoring*

Routine cardiology consultation is not required solely because radioligand therapy is initiated. Cardiovascular investigations should be guided by baseline risk or new symptoms, while complete blood count and renal function should be monitored according to the specific agent. Patients receiving concomitant ADT or ARPIs should follow the cardiovascular monitoring recommendations for those treatments [54].

### 4.4. Immunotherapy

Immunotherapy has revolutionized uro-oncology with several classes now integral to treatment paradigms for bladder, prostate and renal cancers. While therapies like intravesical Bacillus Calmette-Guérin (BCG) remain important in non-muscle invasive bladder cancer, they are associated with minimal systemic and cardiovascular toxicities and thus will not be the focus of this review. The same applies for uro-oncology vaccine therapies, due to localized nature of the immune activation and lower systemic cytokine release.

#### 4.4.1. Immune Checkpoint Inhibitors (ICIs) (Pembrolizumab, Atezolizumab, Nivolumab, Avelumab, Ipilimumab)

##### ICI Monotherapy

ICIs enhance antitumor T-cell responses by targeting PD-1/PD-L1 or CTLA-4. Agents used in urothelial carcinoma and renal cell carcinoma include pembrolizumab, atezolizumab, nivolumab, avelumab, and ipilimumab, depending on the indication and regulatory jurisdiction [56,151].

(a) *Cardiovascular adverse effects*

ICI-associated cardiovascular toxicities include myocarditis, AF, ventricular arrhythmias, conduction disorders, pericarditis, myopericarditis, and HF [152,153,154,155].

A meta-analysis of approximately 59,000 patients reported an increased risk of cardiotoxicity with ICIs (RR 1.31), with myocarditis and arrhythmias predominating [156]. AF is the most frequently reported arrhythmia, followed by ventricular tachycardia and ventricular fibrillation [157].

Overall cardiovascular adverse events occur in approximately 0.8–1.1% of treated patients [55]. Clear agent-specific differences remain insufficiently defined, although combination ICI therapy is generally considered higher risk than monotherapy.

(b) *Mechanisms of cardiovascular toxicity*

By removing inhibitory immune checkpoints, ICIs may activate autoreactive T cells and trigger inflammatory injury involving the myocardium, pericardium, and cardiac conduction system [56,151].

(c) *Clinical significance and long-term cardiovascular risk*

ICI-related myocarditis is uncommon but may be rapidly progressive and is associated with a reported mortality of approximately 38% in meta-analyses and up to 50% in clinical series [158,159,160].

It frequently presents with troponin elevation, new atrioventricular block, or ventricular arrhythmias. Takotsubo syndrome has also been reported, although a causal association with ICI therapy has not been established [161]. Late cardiovascular effects remain less well characterized.

(d) *Role of the cardio-oncologist and monitoring*

Baseline assessment should include ECG and cardiac troponin, with natriuretic peptides and TTE considered according to baseline cardiovascular risk [8].

During treatment, ECG and biomarker surveillance should be risk-adapted, particularly during the early treatment phase. Suspected myocarditis requires immediate ICI interruption, urgent cardio-oncology assessment, and evaluation with ECG, biomarkers, TTE, and cardiovascular magnetic resonance; endomyocardial biopsy should be reserved for selected high-risk or diagnostically uncertain cases [162].

##### Current ICI-TKI Combinations in Renal Cell Carcinoma

Contemporary first-line regimens for advanced renal cell carcinoma include nivolumab plus cabozantinib, pembrolizumab plus axitinib, and pembrolizumab plus lenvatinib [163,164,165,166]. Their cardiovascular profile may reflect additive toxicities from both components, particularly TKI-associated hypertension and ventricular dysfunction together with the uncommon but potentially severe immune-mediated myocarditis and arrhythmias associated with ICIs [8,11]. Cardiovascular surveillance should integrate frequent blood-pressure monitoring with symptom-triggered ECG, troponin, and cardiac imaging.

#### 4.4.2. Chimeric Antigen Receptor (CAR) T-Cell Therapy

CAR-T cell therapy is an emerging, investigational immunotherapeutic modality in uro-oncology (not yet FDA-approved for urologic malignancies), that involves engineering patient T cells to recognize tumor-specific antigens such as prostate-specific membrane antigen (PSMA) and human epidermal growth factor receptor 2 (HER2) [167].

(a) *Cardiovascular adverse effects*

Approximately 16–20% of patients receiving CAR-T therapy develop arrhythmias, cardiomyopathy/HF, myocarditis, and in rare cases, myocardial infarction and cerebrovascular accidents [168,169].

A recent systematic review and meta-analysis also quantified cardiotoxicity after CAR-T cell therapy, reporting a similarly high incidence of arrhythmias (up to 54%), HF (≈30%), and cardiomyopathy (≈20%), with CRS grade ≥ 2 significantly increasing the risk of cardiovascular events [170].

Another pooled analysis across thirteen cohorts showed that LV dysfunction occurred in 8.7% of CAR-T recipients and supraventricular arrhythmias in 7.8%, whereas severe events like myocardial infarction and cardiovascular death were relatively infrequent (<1%), indicating a distinct spectrum of cardiotoxicity in the short- to mid-term [171].

(b) *Mechanisms of cardiovascular toxicity*

Despite its promise, CAR-T therapy carries notable cardiotoxic risks primarily mediated by cytokine release syndrome (CRS), an intense immune activation causing systemic inflammation, that can lead to myocardial depression, vasoplegia-induced hypotension, arrhythmogenesis, and direct cardiac injury due to on-target off-tumor effects on cardiac tissue expressing targeted antigens [57,172].

(c) *Clinical significance and long-term cardiovascular risk*

Cardiovascular events generally occur during the acute or early post-infusion period, and CRS grade ≥ 2 is associated with a higher risk of cardiovascular complications [170]. Uro-oncology-specific and long-term cardiovascular safety data remain unavailable.

(d) *Role of the cardio-oncologist and monitoring*

Patients enrolled in CAR-T trials should undergo baseline cardiovascular risk assessment. During CRS, ECG, cardiac biomarkers, and TTE should be performed according to clinical severity, with early cardio-oncology involvement in patients with pre-existing cardiovascular disease or treatment-emergent cardiac abnormalities [171].

### 4.5. Radiotherapy

Radiotherapy plays an important role in the management of localized and locally advanced prostate, bladder, and penile cancers. Contemporary techniques improve tumor targeting and reduce exposure of surrounding tissues; nevertheless, delayed vascular toxicity remains clinically relevant [58].

(a) *Cardiovascular adverse effects*

Pelvic radiotherapy is associated predominantly with delayed vascular toxicity, including accelerated atherosclerosis, arterial stiffness, and ischemic complications within irradiated vascular territories [59].

HTN may also occur when the renal vasculature is exposed to clinically relevant radiation doses [173].

(b) *Mechanisms of cardiovascular toxicity*

Ionizing radiation induces endothelial injury, oxidative stress, inflammation, DNA damage, and progressive fibrosis within irradiated vessels, thereby promoting vascular dysfunction and atherosclerosis [59].

(c) *Clinical significance and long-term cardiovascular risk*

Radiation-associated vascular complications typically have a prolonged latency and may become apparent years or decades after treatment, supporting long-term cardiovascular risk assessment in survivors [174].

(d) *Role of the cardio-oncologist and monitoring*

Routine cardiology consultation is not required before pelvic radiotherapy in every patient. Baseline and long-term follow-up should focus on cardiovascular risk factors, including BP, lipids, glucose, smoking, and renal function, with vascular imaging or cardiology referral guided by symptoms, abnormal findings, pre-existing vascular disease, or high cumulative radiation exposure [174].

Evidence regarding long-term cardiovascular complications after pelvic radiotherapy remains limited and is predominantly observational. Follow-up should focus on aggressive control of conventional cardiovascular risk factors and symptom-driven evaluation for lower-extremity or aorto-iliac vascular disease. Routine coronary imaging or echocardiographic surveillance schedules derived from thoracic radiotherapy should not be directly applied to asymptomatic patients exposed exclusively to pelvic fields. No evidence-based fixed 5–10-year cardiovascular screening interval has been established for this population [174].

In addition, according to the 2022 ESC Cardio-Oncology Guidelines, renal artery ultrasound should be considered in patients with a history of abdominal or pelvic radiotherapy who develop worsening renal function and/or systemic hypertension (Class IIa, Level C) [8].

## 5. Cardiovascular Risk Assessment, Monitoring, and Prevention in Uro-Oncology

Cancer-therapy related cardiovascular toxicity in uro-oncology represents a critical challenge, particularly given the widespread use of ADT, chemotherapy, targeted therapies, and immunotherapies. These treatments significantly improve cancer outcomes but may induce or exacerbate cardiovascular complications, including IHD, HF, arrhythmias, HTN, and thromboembolism (Table 1).

Effective management begins with comprehensive cardiovascular risk assessment before therapy initiation. Stratification guides treatment choices, balancing oncologic efficacy with cardiovascular safety [166,175]. Clinical evaluation should incorporate validated risk stratification tools, such as SCORE2 (Systematic Coronary Risk Evaluation)/SCORE2-OP (Older Persons) or ASCVD (Atherosclerotic Cardiovascular Disease) Risk Estimator in patients without baseline atherosclerotic disease, alongside thorough history-taking and physical examination [176]. This includes evaluating traditional risk factors, such as HTN, diabetes, dyslipidemia, and previous cardiac events, and utilizing tools like echocardiography and biomarkers (troponins, NT-proBNP) to establish baseline cardiac function [177]. Cardiovascular risk estimation is recommended in patients undergoing cancer therapy with potential to induce CTR-CVT and should be discussed with the treating team, including a cardiology consultation if estimated risk is moderate or high/very high. Early identification of CTR-CVT allows for prompt intervention, potentially mitigating progression to clinical HF or MACE [9].

Prior to initiation of cardiotoxic uro-oncological therapies, cardiovascular risk stratification should be performed using validated tools where available. Therapy-specific HFA-ICOS (Heart Failure Association-International Cardio-Oncology) proformas exist for select agent classes (e.g., anthracyclines, VEGF inhibitors, ICIs) and should be used when applicable [178]; for agents without a validated proforma, individualized guideline-based cardiovascular risk assessment (e.g., SCORE2/SCORE2-OP) should be used instead [179].

Prophylactic strategies should be personalized according to risk profile.

Lifestyle optimization through smoking cessation, dietary patterns improvement, and physical activity is fundamental for comprehensive cardiovascular risk management [180].

Besides beta-blockers and ACE inhibitors, recent evidence supports the use of angiotensin receptor blockers and mineralocorticoid receptor antagonists to reduce cardiac remodeling and oxidative stress associated with cancer therapies [181]. Statins contribute to additional cardiovascular protection by stabilizing atherosclerotic plaques and attenuating inflammation [182]. Dexrazoxane remains a valuable agent in preventing anthracycline-induced cardiotoxicity through its antioxidative properties [183]. Emerging agents, such as sodium-glucose co-transporter 2 (SGLT2) inhibitors, show promise in reducing cardiovascular events and warrant further investigation within this patient population [184].

During therapy, close cardiovascular monitoring is vital to detect early subclinical dysfunction and mitigate adverse outcomes [185]. Periodic evaluation of BP, ECGs, and imaging can identify treatment-related toxicities promptly [186]. Beyond conventional clinical assessment, growing evidence supports the role of multimodal congestion assessment- including biomarkers, echocardiography, lung ultrasound, and venous ultrasound techniques- for earlier identification of subclinical congestion and optimization of cardiovascular management in high-risk cardio-oncology patients [185,187].

A multidisciplinary collaboration among oncologists, cardiologists, urologists and primary care providers is essential for optimal cardiovascular care in uro-oncology patients. Establishing care pathways that allow shared decision-making, streamlined referrals, and coordinated long-term surveillance maximizes both cancer outcomes and cardiovascular health.

**Figure 1:**
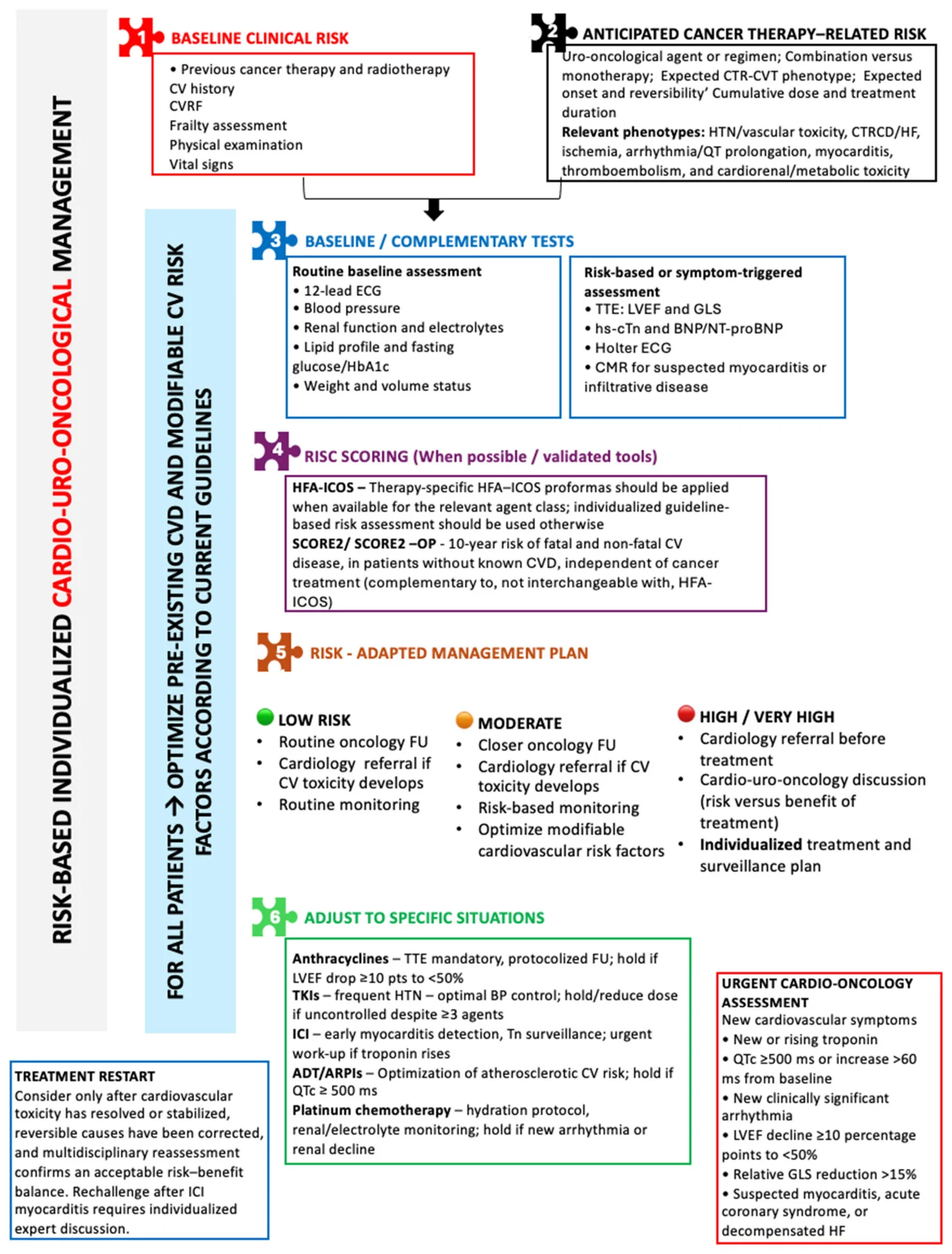
Uro-oncology-specific algorithm for individualized cardiovascular risk assessment and management. The six-step pathway integrates baseline clinical and anticipated treatment-related cardiovascular risk, routine and risk-based investigations, and complementary risk stratification using HFA–ICOS and SCORE2/SCORE2-OP. Therapy-specific HFA–ICOS proformas should be applied when available for the relevant agent class; individualized guideline-based risk assessment should be used otherwise. Management is adapted to the estimated risk category and to the specific uro-oncological therapy. The algorithm also highlights criteria for urgent cardio-oncology assessment and principles for treatment interruption and restart after cardiovascular toxicity. Detailed monitoring schedules are provided in Table 2. Abbreviations: ADT, androgen deprivation therapy; ARPI, androgen receptor pathway inhibitor; BNP, B-type natriuretic peptide; BP, blood pressure; CMR, cardiovascular magnetic resonance; CTR-CD, cancer therapy-related cardiac dysfunction; CTR-CVT, cancer therapy-related cardiovascular toxicity; CV, cardiovascular; CVD, cardiovascular disease; CVRF, cardiovascular risk factors; ECG, electrocardiogram; FU, follow-up; GLS, global longitudinal strain; HbA1c, glycated hemoglobin; HF, heart failure; HFA–ICOS, Heart Failure Association–International Cardio-Oncology Society; hs-cTn, high-sensitivity cardiac troponin; HTN, hypertension; ICI, immune checkpoint inhibitor; LVEF, left ventricular ejection fraction; NT-proBNP, N-terminal pro-B-type natriuretic peptide; QTc, corrected QT interval; SCORE2, Systematic Coronary Risk Evaluation 2; SCORE2-OP, Systematic Coronary Risk Evaluation 2–Older Persons; TKI, tyrosine kinase inhibitor; Tn, troponin; TTE, transthoracic echocardiography.

**Figure 2:**
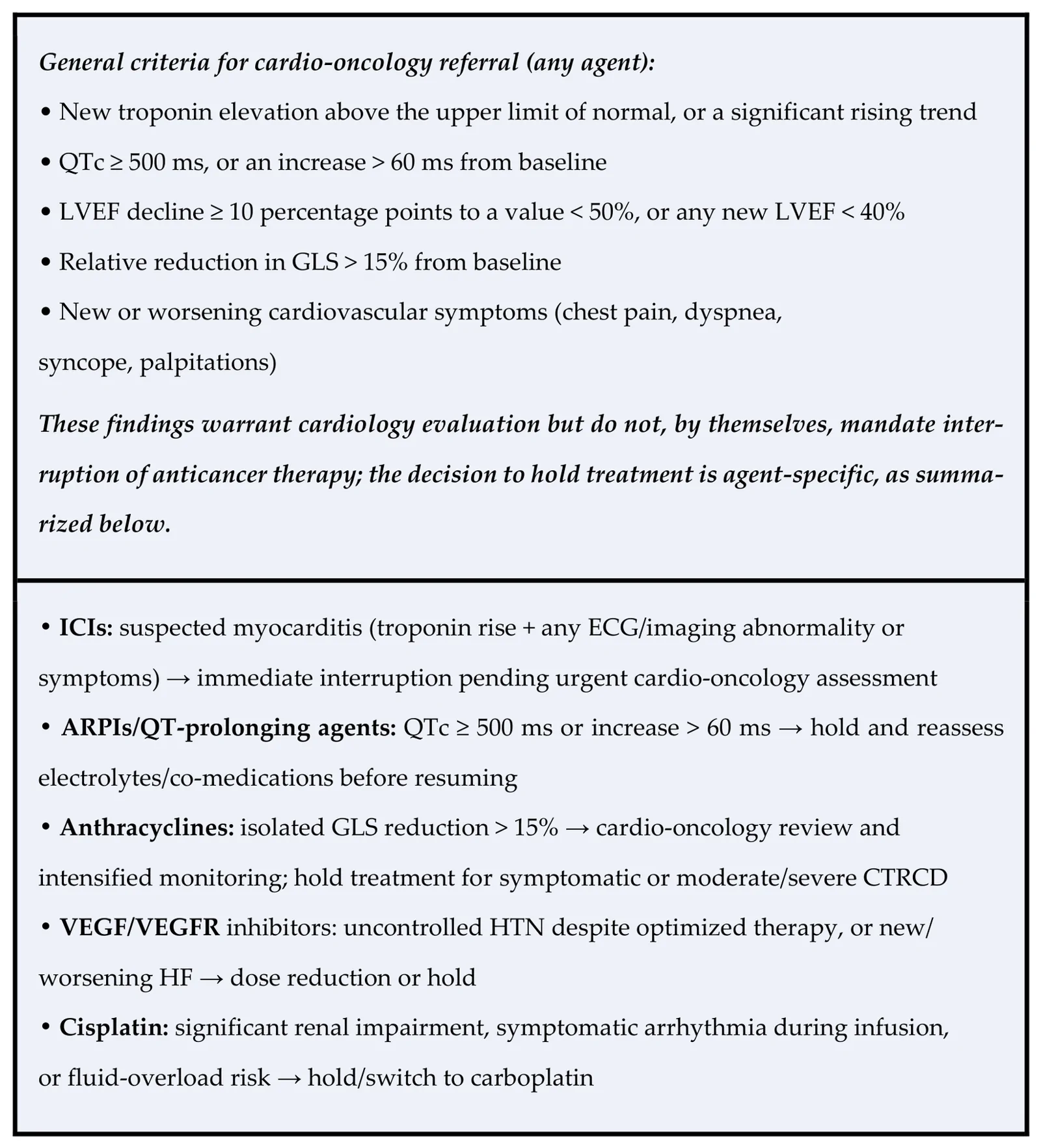
Proposed criteria for cardio-oncology referral and treatment interruption during uro-oncological therapies. Upper panel: general referral criteria applicable to all anticancer agents. Lower panel: selected therapy-specific situations that warrant treatment interruption, dose modification, or urgent cardio-oncology assessment.

Given the heterogeneity of uro-oncological patients and the variability in cardiovascular risk profiles and treatment-related toxicities, a uniform management strategy is not appropriate. Therefore, we propose a structured, stepwise cardio-oncology algorithm that integrates baseline cardiovascular risk assessment, CTR-CVT risk, multimodal cardiac evaluation, and validated risk stratification tools to enable risk-adapted and individualized management. The algorithm follows a six-step approach and is detailed in Figure 1. This framework emphasizes individualized, risk-based decision-making across the entire patient pathway. Figure 2 summarizes the proposed criteria for cardio-oncology referral and the principal therapy-specific situations in which temporary treatment interruption or modification should be considered.

Table 2 below detail the specific baseline, on-treatment, and post-treatment monitoring recommendations that operationalize this algorithm, distinguishing routine, risk-based, and symptom-triggered testing, together with agent-specific schedules for the most commonly used uro-oncological therapies.

Monitoring recommendations presented in this review distinguish routine baseline assessment, risk-adapted surveillance during treatment and symptom-triggered investigations according to current ESC cardio-oncology guidance.

Cardiovascular evaluation criteria and treatment-interruption thresholds are presented separately, as the clinical implications of a given abnormality differ by agent and context.

## 6. Cardiovascular Considerations, Drug Interactions, and Long-Term Survivorship in Uro-Oncology

### 6.1. Supportive Care During Cisplatin-Based Chemotherapy

Platinum-based chemotherapy, especially cisplatin in combination with gemcitabine, is standard of care for muscle-invasive bladder cancer (MIBC) and other advanced urological malignancies. The gemcitabine-cisplatin (GC) regimen is recommended as neoadjuvant therapy for cisplatin-eligible patients with these types of cancers, as supported by leading guideline protocols [188,189]. Cisplatin eligibility is typically defined by the Galsky criteria: ECOG performance status 0–1, glomerular filtration rate ≥ 60 mL/min, Common Terminology Criteria for Adverse Events (CTCAE) grade ≤ 1 hearing loss, grade ≤ 1 peripheral neuropathy, and New York Heart Association (NYHA) class ≤ II heart failure; patients who do not meet these criteria require individualized selection of alternative treatment strategies according to the oncological setting [190]. The Galsky criteria were originally developed to guide cisplatin eligibility in metastatic urothelial carcinoma. Their application to the neoadjuvant MIBC setting is widely adopted in practice but has not been formally validated in this population, and perioperative cardiovascular risk (e.g., surgical fitness, anesthesia tolerance) may not be fully captured by these criteria alone [190].

Beyond standard neoadjuvant GC, two emerging perioperative strategies have shown clinical benefit and warrant cardiovascular awareness. Dose-dense MVAC (methotrexate, vinblastine, doxorubicin, cisplatin; ddMVAC), evaluated in the phase III VESPER trial, improved pathological complete response and 5-year overall survival compared with GC (66% vs. 57%; HR 0.71), but includes doxorubicin, introducing a dose-dependent anthracycline cardiotoxicity risk (see Section 4.2.3) not present with GC alone [41].

Separately, the phase III NIAGARA trial established perioperative durvalumab (an anti-PD-L1 checkpoint inhibitor) combined with neoadjuvant GC, followed by adjuvant durvalumab, as a new standard of care with an overall survival benefit (HR 0.75), adding immune-related adverse event risk—including immune-mediated myocarditis—to the established cardiovascular toxicity profile of cisplatin-based chemotherapy [40].

Given the cumulative cardiovascular burden of these intensified regimens, baseline cardiovascular risk stratification is particularly important when selecting among GC, ddMVAC, and durvalumab-based perioperative strategies.

Effective prevention of cisplatin-induced cardiovascular and renal toxicity in urothelial cancer relies on adherence to international supportive care guidelines [191]. The National Comprehensive Cancer Network (NCCN) and the European Association of Urology (EAU) recommend pre- and post-hydration with intravenous isotonic saline to promote urine output, enhance cisplatin clearance, and reduce nephrotoxicity [192,193]. Standard protocols include 1–2 L of 0.9% saline before cisplatin, followed by an additional 1–2 L after infusion, for a total of 2–4 L over 4–6 h, a strategy shown to reduce acute kidney injury without increasing heart failure risk, even in patients with cardiac dysfunction [191].

Guidelines from eviQ and Cancer Care Ontario recommend adding magnesium sulfate (10–16 mmol) to hydration before and after cisplatin administration to prevent hypomagnesemia, a risk factor for arrhythmias and nephrotoxicity [194]. Some protocols also include potassium chloride (20 mmol) to reduce hypokalemia and maintain cardiac electrical stability [195]. When necessary, mannitol or furosemide may be used to achieve urine output > 100 mL/h, limiting cisplatin exposure to the renal tubules [196]. Nonetheless, aggressive hydration may precipitate fluid overload, pulmonary congestion, or edema in patients with heart failure or uncontrolled hypertension, underscoring the need for careful fluid management in these high-risk patients [69].

Steroids, particularly dexamethasone, are commonly used in chemotherapy protocols primarily for their antiemetic and anti-inflammatory effects rather than for direct cardiovascular protection [197]. Corticosteroids also carry known cardiovascular risks—HTN, dyslipidemia, fluid retention, and arrhythmogenic potential—particularly with prolonged or high-dose regimens [198].

### 6.2. Clinically Relevant Drug–Drug Interactions

Beyond direct cardiotoxicity, several uro-oncological agents carry clinically significant pharmacokinetic interactions that can indirectly increase cardiovascular risk, summarized in Table 3.

### 6.3. Long-Term Cardiovascular Surveillance in Survivors

Cardiovascular risk in uro-oncology extends well beyond active treatment. Cisplatin-based chemotherapy, prolonged androgen deprivation, and pelvic radiotherapy are each associated with long-latency, often irreversible cardiovascular sequelae that warrant dedicated long-term surveillance independent of oncologic follow-up, summarized in Table 4.

## 7. Conclusions

Cardiovascular complications are a significant clinical challenge in patients undergoing uro-oncological treatments, including ADT, chemotherapy, targeted therapies, immunotherapy, and radiotherapy. These therapies contribute to a broad spectrum of cardiotoxicities, such as metabolic disturbances, endothelial dysfunction, arrhythmias, HF, and thromboembolism.

Multidisciplinary collaboration integrating oncology, urology and cardiology expertise is fundamental to optimize patient outcomes, balancing effective urological cancer treatment with cardiovascular health preservation. Ongoing research should focus on enhancing risk prediction, understanding underlying mechanisms, and developing targeted cardioprotective strategies specific to uro-oncology patients.

Future progress in uro-cardio-oncology will rely on individualized cardiovascular risk stratification, multidisciplinary management and survivorship programs specifically addressing long-term cardiovascular complications after contemporary uro-oncological therapies.

## Figures and Tables

**Table 1:** Cardiovascular toxicities of uro-oncological therapies by cancer type, indication, and regimen.

Cancer Type/ Indication	Regimen/Agent	Treatment Status	CV Toxicity	Incidence/ Severity	Onset	Reversibility	Risk Modifiers	Evidence Level	Reference
Prostate cancer (ADT)	GnRH Agonists (Leuprolide, Goserelin)	Approved	Metabolic alterations, HTN, HF, AF, thromboembolism, QT prolongation	Common; exact rates vary across studies	Acute + long-term	Largely reversible on discontinuation	Pre-existing CVD, older age, treatment duration	C	[17,18]
Prostate cancer (ADT)	GnRH Antagonists (Degarelix, Relugolix)	Approved	Possible cardiovascular advantage compared with GnRH agonists, particularly for relugolix in HERO; class-wide reduction in MACE, hypertension, or arrhythmias remains unproven.	HERO reported fewer MACE with relugolix than with leuprolide; PRONOUNCE was neutral and underpowered. Findings from pooled and observational studies remain heterogeneous.	Acute	Reversible	Prior cardiovascular disease (differential benefit)	A	[19,20]
Prostate cancer (ADT)	Surgical castration (Bilateral orchiectomy)	Approved	Ischemic cardiac events, peripheral artery disease	Higher vs. medical castration in first 18 months; similar thereafter	Acute (first 18 months)	Permanent procedure; CV risk elevation transient	Older age, pre-existing CVD	B	[21,22,23]
Prostate cancer (ADT)	First-generation Antiandrogens (Flutamide, Bicalutamide, Nilutamide)	Largely historical; monotherapy abandoned	Lower relative CV risk;	Lower than GnRH agonists	Acute	Reversible	None specific	C	[24]
Prostate cancer (M0-CRPC and beyond)	Second-generation ARPIs (Enzalutamide, Apalutamide, Darolutamide)	Approved	HTN, IHD, AF, HF	HTN up to 30%	Acute + long-term	Largely reversible	Pre-existing HTN/CVD, concurrent ADT	A	[25,26]
Prostate cancer (mCRPC/mHSPC)	Androgen Synthesis Inhibitors (Abiraterone)	Approved	HTN, fluid retention, hypokalemia, HF (mineralocorticoid excess)	Common; dose-related	Acute	Reversible with dose adjustment/MRA	Pre-existing HF, hypokalemia	B	[27]
Prostate cancer (historical)	Ketoconazole, Cyproterone acetate	Largely abandoned/historical	Thromboembolism reported with cyproterone acetate; limited cardiovascular data for ketoconazole	Not well quantified	Acute	Reversible if detected early	Not established	C	[28,29]
Prostate cancer (BRCA/HRRm mCRPC, 1st line)	PARP inhibitor + ARPI combinations (Olaparib+abiraterone)	Approved	No significant additive CV signal (PROpel); VTE/PE risk	CV events not significantly different vs. abiraterone alone	Acute	Reversible	Additional prothrombotic risk factors	A	[30]
Multiple (bladder, testicular, other)	Platinum agents (Cisplatin, Carboplatin, Oxaliplatin)	Approved	HTN, myocardial ischemia, arrhythmias, thromboembolism, Takotsubo	Variable; thromboembolism common acutely	Acute + long-term	Partial (acute reversible; long-term vascular aging irreversible)	Cumulative dose, pre-existing CVD	C	[31,32]
Testicular/penile cancer	Cisplatin-based regimens (BEP, EP, VIP, TIP, TPF, PF)	Approved	Endothelial injury, prothrombotic state, atherosclerosis, Raynaud’s phenomenon	1.4–7-fold long-term CVD risk; MI 2–3-fold (BEP)	Acute (VTE) + tardive (vascular aging)	Largely irreversible (long-term vascular aging)	Cumulative cisplatin dose, smoking, renal decline	B	[33,34,35]
Prostate cancer (mCRPC)	Taxanes (Paclitaxel, Docetaxel, Cabazitaxel)	Approved	LV dysfunction, arrhythmias (tachycardia, AF), ischemic events	Cabazitaxel: cardiac disorders 4.8% vs. 0.8% control; overall estimates vary among taxanes.	Acute	Generally reversible	Older, comorbid mCRPC population	A	[36,37]
Bladder/prostate cancer (less common)	Anthracyclines (Doxorubicin)	Approved	Irreversible cardiotoxicity, dilated cardiomyopathy, HF	HF ~6% at 10 years; subclinical LV dysfunction up to 18%	Tardive; cumulative-dose dependent	Largely irreversible	Cumulative dose, pre-existing cardiac disease	C	[38]
Bladder cancer/other	Antimetabolites (Gemcitabine, 5-Fluorouracil, Capecitabine)	Approved	Coronary vasospasm, angina, MI, arrhythmias	Uncommon but described	Acute	Often reversible on discontinuation	Pre-existing CAD	C	[39]
Bladder cancer (MIBC, perioperative)	ddMVAC; Durvalumab+GC (NIAGARA)	Approved	Durvalumab + GC: MI and PE reported; ddMVAC: potential anthracycline-related CTRCD.	NIAGARA: fatal MI 0.2%, serious PE 1.9%; VESPER did not report a dedicated cardiovascular endpoint	Acute (myocarditis) + tardive (anthracycline)	Partial (myocarditis often reversible if caught early; anthracycline damage largely irreversible)	Cumulative anthracycline dose; pre-existing autoimmune disease (ICI)	A	[40,41]
Renal cell carcinoma/urothelial	VEGF/VEGFR Inhibitors (Sunitinib, Pazopanib, Sorafenib, Axitinib, Cabozantinib, Lenvatinib, Tivozanib)	Approved	HTN, arterial stiffness, LV hypertrophy, HF, QT prolongation	HTN up to 80%; lenvatinib all-grade 50%, grade ≥ 3 14%	Acute (early, first weeks)	Reversible with dose modification	Pre-existing HTN, older age	A	[42,43]
Urothelial carcinoma (FGFR-altered)	FGFR inhibitors (Erdafitinib)	Approved	Hypertension and rare cardiac adverse-event signals	Pharmacovigilance signal; absolute incidence cannot be estimated	Acute	Not established	Not established	C	[44]
Urothelial/other (investigational)	Debio-1347, Dovitinib, Derazantinib	Investigational	HTN, cardiac dysfunction (limited data)	Not well established	Unclear	Unclear	Unknown	C	[45]
Renal cell carcinoma	mTOR inhibitors (Everolimus, Temsirolimus)	Approved	HTN, HF, LV dysfunction	HTN ~24%	Acute	Reversible	Pre-existing HTN	C	[46,47]
Renal cell carcinoma (VHL-associated)	HIF-2α inhibitors (Belzutifan)	Approved	Low CV toxicity;	Low	Acute	Reversible	None specific	A	[48,49]
Prostate cancer (mCRPC, BRCA/HRRm)	PARP inhibitors, monotherapy (Olaparib, Rucaparib, Talazoparib, Niraparib)	Approved	MACE, HTN, arterial/venous thromboembolic events	MACE 5.0% vs. 3.6% control; HTN 17.5%	Acute + ongoing	Partial	Pre-existing thromboembolic risk	A	[50]
Urothelial carcinoma	Enfortumab vedotin (ADC)	Approved	Pharmacovigilance signals for HF, VTE, hypertension, arrhythmias, and QT prolongation	Absolute incidence cannot be estimated from pharmacovigilance data	Acute	Variable	Pre-existing cardiac disease	C	[51]
Urothelial carcinoma (1st line)	Enfortumab vedotin + Pembrolizumab	Approved	No prominent cardiovascular safety signal; potential ICI-related myocarditis and ADC-associated cardiovascular events	Serious cardiac AEs were not among the most frequent events (≥2%); individual cardiac events were <2%	Variable, per component	Variable, per component	Autoimmune disease history (ICI component)	A	[52]
Prostate cancer (mCRPC, bone mets)	Radium-223	Approved	Low direct cardiac signal	MI ~0.2%, AF ~0.4%	Acute	Reversible	Not established	A	[53]
Prostate cancer (mCRPC)	^177^Lu-PSMA-617	Approved	No major direct cardiac signal	Low (long-term CV data limited)	Unclear (long-term)	N/A	Not established	A	[54]
Multiple uro-oncology cancers	Immune Checkpoint Inhibitors (Pembrolizumab, Nivolumab, etc.)	Approved	Myocarditis (high mortality), AF, ventricular arrhythmias, pericarditis, HF	Myocarditis rare but high mortality in reported series	Acute; potentially fulminant	Variable; can be irreversible if fulminant	Combination ICI therapy, pre-existing autoimmune disease	A	[55,56]
Uro-oncology (investigational)	CAR-T Cell Therapy	Investigational (uro-oncology)	CRS-induced myocardial depression, hypotension, arrhythmias, myocarditis	~16–20% develop cardiotoxicity	Acute (during/after infusion)	Often reversible with CRS management	High CRS grade, pre-existing cardiac disease	C	[57]
Prostate/bladder cancer	Pelvic Radiotherapy	Approved	Long-term cardiovascular events and heart-specific mortality; possible pelvic-field peripheral/aorto-iliac vascular disease	Observational evidence; absolute risks vary, and no fixed cardiovascular screening interval has been established	Tardive (long latency)	Largely irreversible	Radiation dose/field, pre-existing CVD	B	[58,59]

ADT: androgen deprivation therapy; ARPI: androgen receptor pathway inhibitor; ADC: antibody-drug conjugate; AF: atrial fibrillation; CAR-T: chimeric antigen receptor T-cell; CRS: cytokine release syndrome; CV: cardiovascular; CVD: cardiovascular disease; HF: heart failure; HTN: hypertension; ICI: immune checkpoint inhibitor; IHD: ischemic heart disease; MACE: major adverse cardiovascular events; M0-CRPC: non-metastatic castration-resistant prostate cancer; mCRPC: metastatic castration-resistant prostate cancer; mHSPC: metastatic hormone-sensitive prostate cancer; MI: myocardial infarction; MIBC: muscle-invasive bladder cancer; MRA: mineralocorticoid receptor antagonist; VTE: venous thromboembolism. Evidence level refers to the evidence supporting the cardiovascular outcome: A, randomized trial or meta-analysis reporting cardiovascular data; B, prospective cohort, registry, or cardiovascular safety analysis from a large oncology trial; C, retrospective study, pharmacovigilance analysis, case series, review, or expert opinion.

**Table 2:** General cardiovascular monitoring framework across uro-oncological therapies (a). Agent-specific cardiovascular monitoring schedules (b).

(**a**)			
**Parameter**	**Baseline**	**On-Treatment**	**Post-Treatment/** **Survivorship**
ECG (incl. QTc)	Routine—all patients starting cardiotoxic or QT-prolonging therapy	Risk-based (QT-prolonging agents, VEGF inhibitors) or symptom-triggered (palpitations, syncope)	Symptom-triggered
TTE (LVEF + GLS)	Routine—all patients planned for anthracyclines, HER2-independent cardiotoxic regimens, or with pre-existing CVD	Risk-based per agent/cumulative dose (e.g., every 2–3 cycles for anthracyclines) or symptom-triggered	Risk-based—periodic surveillance in high-risk survivors (anthracycline-exposed, testicular cancer survivors)
Troponin/Natriuretic peptides (NT-proBNP)	Routine at baseline for patients receiving ICIs; risk-based for anthracyclines and other therapies according to baseline cardiovascular risk	Risk-adapted according to agent and baseline risk; see Table 2(b) for the ICI schedule	Symptom-triggered
Blood pressure/Home BP monitoring	Routine—all patients	Routine—weekly during VEGF-inhibitor dose titration, then periodic; routine with ADT/abiraterone/ARPIs	Routine, periodic
Electrolytes (K^+^, Mg^2+^)	Routine	Routine with abiraterone/mineralocorticoid-excess agents; risk-based otherwise	Symptom-triggered
Renal function	Routine	Routine—each cycle with cisplatin; periodic with other nephrotoxic agents	Routine, periodic (particularly cisplatin-treated survivors)
Weight/Volume status	Routine	Routine—agents associated with fluid retention (ARPIs, VEGF inhibitors)	Symptom-triggered
Metabolic tests (fasting glucose, lipid panel)	Routine	Periodic—ADT, mTOR inhibitors	Routine, periodic (long-term ADT and testicular cancer survivors)
(**b**)			
**Agent/Regimen**	**Baseline**	**On-Treatment**	**Post-Treatment**
ADT (GnRH agonists/antagonists, orchiectomy)	ECG, BP, fasting glucose, lipid panel, weight	BP and metabolic panel every 3–6 months	Annual cardiovascular risk reassessment
Abiraterone	BP, serum potassium, liver function tests	BP and potassium weekly for first month, then monthly; LFTs monthly for first 3 months	Periodic BP/electrolyte monitoring
Niraparib + Abiraterone	BP, complete blood count, potassium	BP weekly for first month then monthly; CBC monthly for first year (VTE and cytopenia vigilance)	Periodic BP/CBC monitoring
Cisplatin-based regimens	ECG, renal function, magnesium/potassium, TTE if additional risk factors	Renal function each cycle; ECG if symptomatic; monitor for arrhythmia during infusion	Long-term CV risk assessment (BP, lipids, glucose) in survivors, particularly testicular cancer
VEGF/VEGFR inhibitors	BP, TTE/LVEF, ECG (QTc)	Home BP weekly during first cycle then periodic; LVEF at ~3 months or if symptomatic	Periodic BP monitoring
Immune checkpoint inhibitors (ICIs)	ECG, troponin (routine); TTE/LVEF risk-based (per baseline CV risk)	ECG and troponin before doses 2–4; thereafter risk-adapted surveillance, with immediate assessment if symptoms or abnormalities occur.	Symptom-triggered; consider continued vigilance through first year
Anthracyclines	TTE/LVEF + GLS, troponin	GLS and troponin surveillance per cycle in high-risk patients; cumulative dose tracking	Long-term LVEF surveillance, especially in young/testicular or bladder cancer survivors

Testing intensity is classified as routine (recommended for all patients), risk-based (guided by baseline cardiovascular risk, agent, or cumulative dose), or symptom-triggered (performed only in response to new cardiovascular symptoms). BP: blood pressure; CVD: cardiovascular disease; GLS: global longitudinal strain; ICI: immune checkpoint inhibitor; LVEF: left ventricular ejection fraction; NT-proBNP: N-terminal pro-B-type natriuretic peptide; TTE: transthoracic echocardiography. ARPI: androgen receptor pathway inhibitor; CBC: complete blood count; LFTs: liver function tests; VTE: venous thromboembolism.

**Table 3:** Clinically relevant drug–drug interactions affecting cardiovascular risk in uro-oncology.

Agent/Class	Interacting Drug/Class	Mechanism	Clinical Consequence	Management
Enzalutamide (ARPI)	CYP3A4/P-gp substrates (e.g., warfarin, many statins, DOACs)	Strong CYP3A4 and moderate CYP2C9/CYP2C19 induction	Reduced plasma levels and efficacy of interacting drug (e.g., subtherapeutic anticoagulation, thrombosis risk)	Avoid combination where possible; Anticoagulant selection should be individualized according to the specific substrate, renal function, thrombotic and bleeding risk, and availability of drug-level or INR monitoring. Routine substitution of a DOAC with warfarin is not necessarily safer because warfarin exposure may also be reduced by enzyme induction.
Apalutamide (ARPI)	CYP3A4 substrates (e.g., many statins, some DOACs)	Strong CYP3A4/CYP2C19, weak CYP2C9, and weak P-gp/BCRP/OATP1B1 induction	Reduced plasma levels and efficacy of interacting drug	Same principles as enzalutamide apply for CYP3A4 substrates; P-gp-mediated DOAC interaction may be less pronounced, but individualized monitoring is still recommended given limited comparative data
Abiraterone	Strong CYP3A4 inducers/inhibitors; CYP2D6 substrates (e.g., some antiarrhythmics)	Abiraterone metabolism altered by CYP3A4 modulators; weak CYP2D6 inhibition by abiraterone	Altered abiraterone exposure; increased plasma levels of CYP2D6 substrates with narrow therapeutic index	Avoid strong CYP3A4 inducers; use caution and consider dose adjustment with CYP2D6 substrates
ADT (all forms) and ARPIs	QT-prolonging drugs (Class IA/III antiarrhythmics, certain antipsychotics, macrolides, fluoroquinolones)	Testosterone suppression itself prolongs QTc; additive effect with other QT-prolonging agents	Increased risk of torsades de pointes	Baseline and on-treatment ECG/QTc; avoid unnecessary co-administration of QT-prolonging drugs
Cisplatin	Nephrotoxic or ototoxic drugs (aminoglycosides, loop diuretics, NSAIDs)	Additive nephrotoxicity/ototoxicity; reduced cisplatin clearance	Increased risk of acute kidney injury, which may secondarily impair cardiovascular status	Avoid combination when possible; monitor renal function closely during and after cisplatin cycles

ADT: androgen deprivation therapy; ARPI: androgen receptor pathway inhibitor; BCRP: breast cancer resistance protein; CYP: cytochrome P450; CYP2D6: cytochrome P450 2D6; DOAC: direct oral anticoagulant; ECG: electrocardiogram; INR: international normalized ratio; NSAID: non-steroidal anti-inflammatory drug; OATP1B1: organic anion transporting polypeptide 1B1; P-gp: P-glycoprotein; QTc: corrected QT interval.

**Table 4:** Long-term cardiovascular surveillance in uro-oncology survivors.

Exposure	Survivor Population	Long-Term Cardiovascular Risk	Recommended Long-Term Surveillance
Cisplatin-based chemotherapy (BEP, EP, VIP, TIP, TPF, PF)	Testicular and penile cancer survivors	1.4–7-fold higher long-term CVD risk; 2–3-fold higher MI risk (BEP); accelerated vascular aging	Annual cardiovascular risk factor assessment (BP, lipid panel, fasting glucose); consider baseline and periodic vascular assessment in long-term survivors, particularly beyond 10–20 years post-treatment
Prolonged androgen deprivation therapy (>1 year)	Prostate cancer survivors	Increased MACE, metabolic syndrome, and heart failure risk with treatment duration	Annual cardiovascular risk reassessment; periodic metabolic monitoring (glucose, lipids); cardiology referral if intermediate/high baseline risk
Pelvic radiotherapy	Prostate and bladder cancer survivors	Accelerated atherosclerosis, arterial stiffness, microvascular ischemia; long latency period (years to decades)	Periodic assessment of conventional cardiovascular risk factors; targeted vascular evaluation in the presence of claudication, diminished peripheral pulses, vascular bruits, resistant hypertension, or other suggestive findings. Routine cardiac imaging schedules derived from thoracic radiotherapy are not recommended for patients treated exclusively with pelvic radiotherapy. Renal artery ultrasound should be considered in patients with a history of abdominal or pelvic radiotherapy who develop worsening renal function and/or systemic hypertension (ESC Class IIa, Level C).
Anthracycline-containing regimens (e.g., ddMVAC)	Bladder cancer survivors	Cumulative dose-dependent risk of heart failure and left ventricular dysfunction	Periodic LVEF surveillance proportional to cumulative anthracycline dose, particularly with additional cardiotoxic treatment exposures

BP: blood pressure; CVD: cardiovascular disease; ddMVAC: dose-dense methotrexate, vinblastine, doxorubicin, cisplatin; LVEF: left ventricular ejection fraction; MACE: major adverse cardiovascular events; MI: myocardial infarction.

## Data Availability

No new data were created or analyzed in this study. Data sharing is not applicable to this article.

## Abbreviations

The following abbreviations are used in this manuscript:

5-FU	5-Fluorouracil
ACE	angiotensin-converting enzyme
ADCs	Antibody-drug conjugates
ADT	androgen deprivation therapy
AR	androgen receptor
ARBs	angiotensin receptor blockers
ARPIs	androgen receptor pathway inhibitors
ASCVD	Atherosclerotic Cardiovascular Disease
AT1R	angiotensin II type 1 receptor
AUA	American Urological Association
BCG	Bacillus Calmette-Guérin
BEP	Bleomycin, Etoposide, Cisplatin
BP	Blood pressure
CAR	Chimeric Antigen Receptor
CRS	cytokine release syndrome
CSF1R	colony-stimulating factor 1 receptor
CTCAE	Common Terminology Criteria for Adverse Events
CTLA-4	Cytotoxic T-Lymphocyte Antigen 4
CTR-CVT	Cancer therapy-related cardiovascular toxicity
CTR-CD	Cancer therapy-related cardiac dysfunction
EAU	European Association of Urology
ECG	electrocardiography
EP	Etoposide, Cisplatin
ESC	European Society of Cardiology
FAERS	Food and drug administration Adverse Event Reporting System
FDA	Food and drug administration
FGFR	fibroblast growth factor receptor
GC	gemcitabine-cisplatin
GLS	Global longitudinal strain
GnRH	gonadotropin-releasing hormone
HDL	high-density lipoprotein
HER2	human epidermal growth factor receptor 2
HFA-ICOS	Heart Failure Association-International Cardio-Oncology Society
HIF-2α	Hypoxia-inducible factor 2-alpha
HTN	hypertension
ICI	Immune Checkpoint Inhibitor
ICOS	International Cardio-Oncology Society
IHD	Ischemic heart disease
LDL	low-density lipoprotein
LV	Left ventricle
LVEF	Left ventricle ejection fraction
MACE	major adverse cardiovascular events
mCRPC	metastatic castration-resistant prostate cancer
mHSPC	Metastatic hormone-sensitive prostate cancer
MIBC	muscle-invasive bladder cancer
mTOR	Mammalian target of rapamycin
MVAC	methotrexate, vinblastine, doxorubicin, cisplatin
NCCN	National Comprehensive Cancer Network
NO	Nitric oxide
NOX2	NADPH oxidase
NYHA	New York Heart Association
PARP	Poly (ADP-ribose) polymerase
PD-1	Programmed cell death protein 1
PD-L1	Programmed death-ligand 1
PF	Cisplatin, 5-Fluorouracil
PSMA	prostate-specific membrane antigen
RT	Radiotherapy
SCORE2	Systematic Coronary Risk Evaluation 2
SCORE2-OP	Systematic Coronary Risk Evaluation 2—Older Persons
SGLT2	sodium-glucose co-transporter 2
TIP	Paclitaxel, Ifosfamide, Cisplatin
TKIs	Tyrosine kinase inhibitors
TPF	Docetaxel/Paclitaxel, Cisplatin, 5-Fluorouracil
VEGF	vascular endothelial growth factor
VIP	Etoposide/Vinblastine, Ifosfamide, Cisplatin
VTE	venous thromboembolism
